# Abundant Small Genetic Alterations after Upland Cotton Domestication

**DOI:** 10.1155/2018/9254302

**Published:** 2018-12-18

**Authors:** Ying Bao, Xia Zhang, Xin Xu

**Affiliations:** School of Life Sciences, Qufu Normal University, Qufu, Shandong 273165, China

## Abstract

Domestication has long been recognized as the most direct and effective way to intentionally influence morphological and physiological phenotypes in plants and animals. Consequently, understanding how small genetic alterations contribute to domestication is of considerable importance. In this study, we resequenced the genome of the wild upland cotton variety* Gossypium hirsutum *var.* yucatanense*, the putative wild ancestor of cultivated upland cotton, and then compared single nucleotide polymorphism (SNP) and short insertion and deletion (InDel) variations of the genome with the cultivated accession (TM-1) of* G. hirsutum*. We found approximately 6.6 million SNPs and 0.7 million InDels between the two genomes. Most of the small genetic variations were anchored in the noncoding regions. With regard to potential coding genes, we found 24,035 genes with nonsynonymous SNPs. Interestingly, 2603 genes in domesticated cotton are found that have changed the positions of stop codons or shifted reading frames from that in* G. hirsutum* var.* yucatanense*. This suggests that domestication may have been selected for mutations that restored gene function or that wild cotton has undergone a number of gene inactivation events since its divergence from cultivated cotton. The former scenario seems most likely due to the intense selective pressure applied during the domestication process. These results demonstrate that, within a relatively short period of time, the cotton genome has been readjusted through small genetic changes. The current study provides useful clues for seeking interesting genes for cotton improvement.

## 1. Introduction

As a special evolutionary process, domestication has long been thought of as a logical and effective way to intentionally influence morphological and physiological phenotypes of plants and animals [1–3]. It is believed that human-induced artificial selection, through domestication, is usually strong [4] and changes in traits can occur over a very short time frame [3]. Obviously, relatively large variations in genomic structure, e.g., gain or loss of long-fragment sequences, large-scale chromosomal rearrangements, and gene copy number variations, can usually produce significant genetic consequences by directly affecting the function or expression of target genes [5–7]. For example, in sunflower, a 999-bp upstream insertion in the promoter region of the gene* HaCYC2c* changed the tissue-specific expression pattern of the gene, resulting in a garden variety with disc floret bilaterality [5]. Nevertheless, many agricultural traits in crops are derived from small genetic changes in one or more genes. In rice, at least three genes (*OsCPL1*,* qSH1,* and* SH4*) are confirmed to be involved in the loss of seed-shattering; in this example, all three gene mutations are single nucleotide mutations [8–10]. Similarly, the origin of naked kernels in maize was also found to be the result of a single nucleotide substitution in gene* tga1* [11]. In addition to variations in protein-coding regions, small mutations in noncoding regions also play an important role in domestication [12–16]. It is believed that the small mutations that reside in* cis*-acting regulatory elements (CAREs) often contribute more to domestication because of a lack of detrimental pleiotropic effects [12, 15]. Recently, Sahu and Chattopadhyay [17] used single nucleotide polymorphism (SNP) and short insertion or deletion (InDel) mining of wild and cultivated tomato genomes to reveal a broad-spectrum genetic base in wild tomato species, and erosion of that base in cultivated tomato, suggesting genome-wide adjustments during recurrent selection for agronomically important traits.

Cotton from* Gossypium* L. species has been a natural fiber source for textiles in the New World for approximately 5,000 years [18, 19], and archaeological data also show that the use of cotton in the Old World may date back to the sixth millennium BC [20]. From a phylogenetic point of view, the genus* Gossypium *comprises more than 50 species. Four species, including two from the Americas (*G. hirsutum *and* G. barbadense*) and two from Africa-Asia (*G. arboreum* and* G. herbaceum*), have been domesticated independently from their wild relatives [19, 21]. Among these species,* G. hirsutum*, i.e., upland cotton, accounts for more than 90% of the global market share for cotton production [22].* G. hirsutum* is an allotetraploid species, with the genome AADD, and is believed to have been domesticated in the northern Yucatan, Mexico, from the local wild variant “*yucatanense*” [22–24]. Due to its restricted geographical distribution, self-pollination, lacking of intense natural selection, and limited evolutionary time, wild upland cotton “*yucatanense*” should sustain the most ancestral genetic traits. Therefore this wild variant could serve as an excellent genetic baseline for its cultivated counterparts.

In this study, to trace the footprints of small genetic alterations in the genome of cultivated* G. hirsutum* after domestication, we investigated the genetic status of* G. hirsutum *var.* yucatanense* at the whole genome-wide level and compared it to the published sequence of cultivated upland cotton accession (TM-1)[25], using next-generation sequencing. Our aim is to detect how many small genetic divergences have occurred in the cotton genome within a very short time scale (ca. 5,000 years), and to understand the potential significance of the genetic variations for cotton improvement.

## 2. Materials and Methods

### 2.1. Plant Materials

Seeds of* Gossypium hirsutum* var.* yucatanense* (Accession no. Tx2090, Tx2094, and Tx2095) used in this study were kindly provided by Prof. Jonathan Wendel of Iowa State University. Seed coats were removed and germinated in a culture dish with wet filter paper at 28°C for about 2-5d. The germinated seeds were then planted into some small pots for 20d, and the young seedlings were transferred to an open field in the greenhouse of Qufu Normal University.

### 2.2. DNA Extraction, Libraries Construction, and Sequencing

Fresh young leaves of three individual plants of each accession were harvested. DNAs of three plants of each accession pooled were extracted using EasyPure Plant Genomic DNA Kit (TransGen Biotech, China) following the manufacturer's protocol. After quality assessment, the genomic DNA was randomly sheared to ~350 bp fragments and separated by gel electrophoresis. The purified 350 bp DNA fragments were used to construct DNA libraries using the TruSeq DNA Library Prep Kit (Illumina, USA) in accordance with the manufacturer's protocol. The library was sequenced on an Illumina HiSeq 2000 platform (Illumina, USA) by Novogene Bioinformatics Institute, Beijing, China.

### 2.3. Reads Filtering and Mapping

Paired-end reads (2 x 150 bp) were filtered to remove adapters and low quality reads. If the sequencing reads had more than 10% ambiguous bases and one end of the reads had more than 50% low quality bases (quality value ≤ 5), the reads were removed. 

Clean reads were then assembled and mapped to the reference genome of the cultivated upland cotton TM-1 [25] using Burrows-Wheeler Aligner [26] with parameters at “mem -t 4 -k 32 –M.” Duplicated reads were removed and coverage values were calculated using Samtools [27] and PICARD (http://picard.sourceforge.net).

### 2.4. Variant Detection and Annotation

The raw SNP or InDel (< 50 bp) sets were called by Samtools with parameters at “-q 1 -C 50 -m 2 -F 0.002 -d 1000.” The identified SNPs and InDels were filtered using the following criteria: mapping quality >20 and depth of the variant position >4. Functional annotation of variants was performed by ANNOVAR [28].

## 3. Results

### 3.1. Abundant Small Genetic Divergences in the Wild Cotton Genome

We obtained a total of 137.15 Gb of raw data from the resequencing of* G. hirsutum *var.* yucatanense*. After removal of poor quality sequences, the data included approximately 913 million reads with a guanine-cytosine (GC) content of 36.45%. A large majority of the reads (897,011,816 reads; 98%), could be mapped to the reference genome of cultivated cotton (Acc. TM-1), with an average depth of 51.37X. Comparative genomics of both cotton samples indicated a whole genome-wide genetic divergence of cultivated cotton from wild cotton (Table 1).

**Figure 1:**
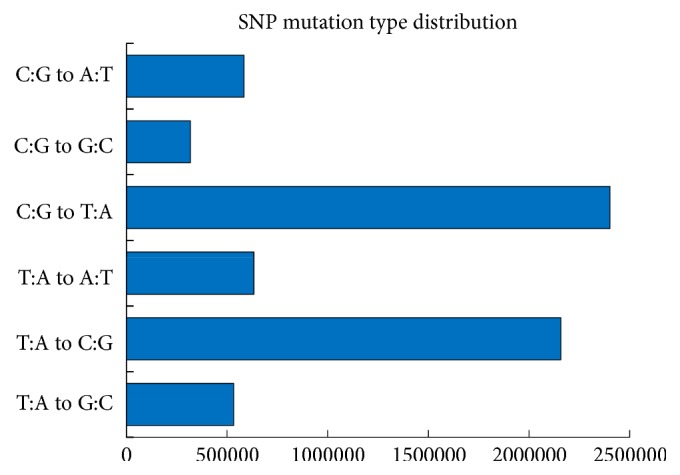
SNP mutation type distribution. x-axis: mutation number; y-axis: six mutation types.

Single nucleotide polymorphisms were widespread between* G. hirsutum* var.* yucatanense *and* G. hirsutum *acc. TM-1. We detected a total of 6,628,900 SNPs, with 951,400 transitions and 5,677,500 transversions. There were six substitution types, and the transversions (C:G to T:A, and T:A to C:G) were most common (Figure 1). In the exonic regions, we found a total of 132,271 SNPs, but only 83,197 were homozygous. Among the homozygous SNPs, 35,603 (in a total of 21,225 genes) were synonymous, 46,762 (occurring in 24,035 genes) were nonsynonymous, and 832 (in 790 genes) in the wild cotton would give rise to terminator mutations (gain or loss stop codon). Of these genes, 356 were A subgenome homoeologs, 394 were D subgenome homoeologs, and 40 lacked subgenome-specific SNPs and thus could not be assigned to a subgenome (Table S1). In the noncoding regions, we found 6,496,629 single nucleotide differences between the two cotton genomes. The richest SNP divergences emerged in the intergenic regions, and there were almost equal numbers of differences in the upstream and downstream regions (281,169) and the intronic regions (283,859) (Table 1).

**Figure 2:**
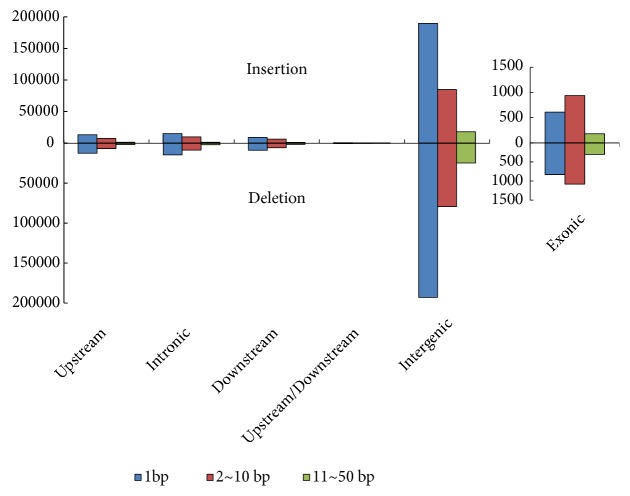
Length distributions of InDel variations in different genome regions. Up: length distributions of insertion variations; down: length distributions of deletion variations.

We also identified a total of 722,586 InDels between the two cotton genomes with roughly equal numbers of insertion (360,033) and deletion (362,554) events (Figure 2). Most of the identified InDels were found in noncoding regions, including 589,730 in the intergenic regions, 77,371 in the upstream and downstream regions, and 51,547 in the intronic regions (Table 1, Figure 2). In contrast, only 3,952 InDels were found in the exonic regions; of these, 2,540 were homozygous. Among the homozygous InDels, 1,389 and 60, in 1,350 genes, had caused protein reading frame shifts or terminator mutations in the wild cotton genome, respectively (Table S2). The majority of the InDel length distributions were shorter than 10 bp; 2 bp-10 bp (especially 3, 6, and 9 bp) and 1 bp were most common in the exonic and noncoding regions, respectively (Figure 2).

### 3.2. Asymmetrical and Fluctuant Subgenomic Genetic Variations after Cotton Domestication

**Figure 3:**
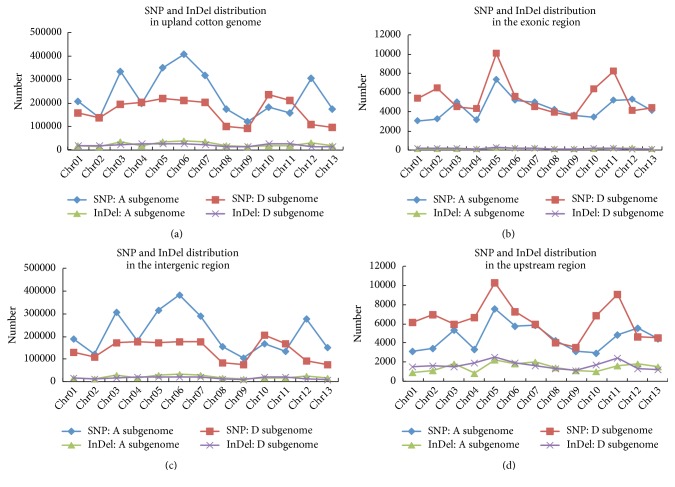
Distribution of genetic variations across the cotton genomes. (a) SNP and InDel distributions in the upland cotton genome; (b) SNP and InDel distributions in the intergenic regions; (c) SNP and InDel distributions in the exonic regions; (d) SNP and InDel distributions in the upstream regions. x-axis indicates chromosome; y-axis indicates the number of SNPs or InDels.

By comparing* G. hirsutum* var.* yucatanense* and* G. hirsutum* accession TM-1, we found that the respective A subgenomes possessed more genetic differences (3,068,445 SNPs and 327,910 InDels) than the respective D subgenomes (2,171,227 SNPs and 278,446 InDels) (Figure 3(a)). This seemed a reasonable finding as that subgenome A is larger than subgenome D [25]. However, when considering the distribution of the variations, an inconsistent and variable subgenome bias emerged. In the exonic coding regions, 39,039 and 42,684 SNPs, and 1,626 and 1,977 InDels, were found in the A and D subgenomes, respectively, suggesting a higher level of coding sequence mutation in the D subgenome compared to the A subgenome (Figure 3(b)). In the noncoding regions, this asymmetrical subgenomic genetic bias began to change. In the intergenic region, a variation preference for A subgenome was observed; a total of 2,791,887 SNPs and 270,464 InDels, and 1,813,542 SNPs and 214,458 InDels, were detected in A and D subgenomes, respectively (Figure 3(c)). In contrast, small genetic variations in the upstream regions of D subgenome (82,127 SNPs and 21,949 InDels) were obviously more abundant than those in A subgenome (59,717 SNPs and 19,240 InDels) (Figure 3(d)). Except for a few cases (e.g., chromosomes 10, 11, and 12), the variation distributions in the different chromosomes followed a general trend; specifically, A subgenome has more nucleotide changes in the intergenic regions and D subgenome has more genetic mutations in the exonic and upstream regions (Figure 3).

## 4. Discussion

### 4.1. Coding Gene Innovation, Reassembly, and Amplification after Domestication

**Figure 4:**
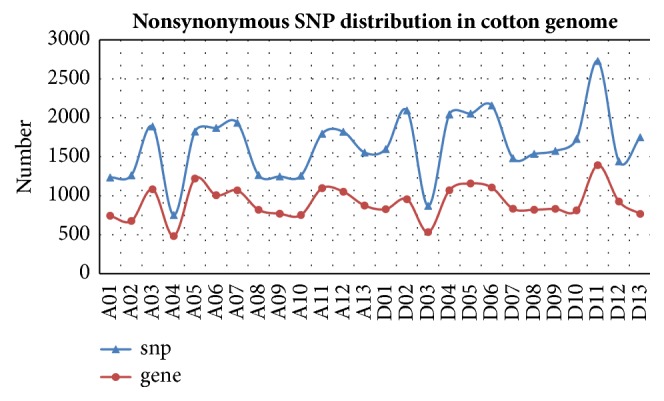
Nonsynonymous SNP distributions in the cotton genome. x-axis indicates chromosome; y-axis indicates the number of nonsynonymous SNPs.

During domestication, cultivated crops have usually undergone strong and recurrent selections and thereby have “footprints” of genetic alterations in their genomes. Specifically, mutations in coding genes are often considered to be an effective way to improve crops [8, 9, 11, 29]. In this study, we revealed 24,035 genes with nonsynonymous nucleotide substitutions between the two cotton genomes. As shown in Figure 4, chromosome D11 had the highest number of nonsynonymous mutations (2,729 in 1,393 genes), and chromosome A04 had the lowest number of nonsynonymous mutations (753 in 483 genes). Previous studies have confirmed that even one amino acid substitution can trigger important agricultural traits in crops [8, 9, 11]. Whether or not the current substitutions are related to morphological or physiological changes involved in cotton domestication will require additional functional studies.

Notably, we also detected that 790 and 1,357 genes changed their stop-codon positions or shifted their open reading frames between the cultivated and wild cottons (Table S1 and S2). Using gene ontology (GO) analysis [30], we found a total of 1,307 function annotations: 831 were categorized into “Molecular function,” 278 into “Biological process,” and 198 into “Cellular component.” In addition, “Protein binding,” “Membrane,” and “Protein tyrosine kinase activity” were the top three GO terms for the genes. “Protein binding” refers to the function of interacting, selectively and noncovalently, with any protein or protein complex; “Membrane” genes provide the media for all the proteins and protein complexes; and “tyrosine kinase activity” refers to the ability to transfer a phosphate group from ATP to a protein and therefore plays an important role in communication signals and regulating cellular activity [6]. These annotation results show that these genes are important in protein interactions, signal transduction, and transcriptional regulation. However, the differences in terminator positions and frame shifts between the* yucatanense* and TM-1 could be a result of either gene elimination in* yucatanense*, or gene/transcript reactivation in TM-1. To investigate whether gene inactivation occurred before or after formation of the* G. hirsutum yucatanense* tetraploid, we used BLAST (https://www.ncbi.nlm.nih.gov/) to compare the 750 loss-of-function genes (356 A subgenome homoeologs and 394 D subgenome homoeologs) to their homologous DNA fragments in the diploid genomes of* G. arboreum* (AA genome; taxid:29729) and* G. raimondii* (DD genome; taxid:29730), respectively. We found that 34% (122 genes) and 28% (113 genes) SNP variations in* yucatanense* were shared by* G. arboreum* and* G. raimondii*, respectively. Therefore, at least for the 235 genes, the SNP mutations in TM-1 ostensibly restored some function to genes that were inactivated prior to the formation of the AADD* G. hirsutum yucatanense.*

### 4.2. Noncoding Variations May Possess Functional Significance

Domesticated crops arose due to both conscious human decision-making and unconscious selection dynamics [31]. Therefore, although crop domestication aims to select traits of interest, the process of artificial selection is intensive and complicated. Upland cotton is thought to have been domesticated from a local Mexican wild variant,* G. hirsutum *var.* yucatanense*, about 5,000 years ago [22–24]. Notably, in such a short period of time, domesticated upland cotton shows extensive noncoding divergence from its wild relative. According to previous studies [25, 32, 33], the size of noncoding sequences is approximately 9 times larger than that of annotated genes in the cotton genome. However, in this study, we revealed approximately 49 and 182 times more SNP and InDel mutations, respectively, in the noncoding regions than in the exonic regions (Table 1). It is true that the noncoding regions can usually avoid strict purifying selection and accumulate greater genetic diversity. However, noncoding alterations, especially the mutations in CAREs, may result in changes in expression of genes and then have important effects on domestication. After assaying genome-wide* cis* and* trans* regulatory differences between maize and teosinte, Lemmon, Bukowski, Sun, and Doebley [34] reveal that genes with* cis*-effects correlated strongly with genes under positive selection during maize domestication and improvement. In cotton, previous studies on whole transcriptomes or special gene families [35–37] showed that the gene expressions between wild and cultivated varieties have been largely reprogrammed, indicating that regulatory changes have played a very important role during upland cotton domestication.

To investigate the potential* cis*-effects of the noncoding variations, based on previous studies [25, 35, 37], we randomly selected five gene loci that possessed significant expression divergence between cultivated and wild cottons, including* Glycerol-3-phosphate acyltransferase 3*,* O-methyltransferase 1*,* Profilin 1*, and two D subgenome homoeologs (*Aldehyde dehydrogenase 7*,* Tyrosine transaminase family protein*). We then compared and predicted the potential CARE variations in the upstream and downstream 2-kb regions of all the above homoeologs using the tool PlantCARE [30]. As a result, we identified a total of 75 SNPs and 33 InDels from the selected noncoding regions of five genes (or eight homoeologs). Among these mutations, we found that 67% of the SNPs, and 63% of the InDels, occurred in regions that related to at least one kind of CARE, and 90% and 52% of these CAREs had been changed in the cultivated cotton in comparison with its wild counterpart (Table S3). In fact, many previous studies have verified that some mutations in noncoding regions could produce divergent CAREs, and these CAREs are usually linked to candidate genes that are related to key agricultural traits and control epigenetic changes in these genes [16, 38, 39]. Recently, using the method of DNase-seq, Wang et al. [15] investigated the active CAREs in cultivated upland cotton and found that approximately half of the CAREs occurred in the promoter and intergenic regions, suggesting the importance of* cis*-regulation in noncoding regions.

Considering that wild and cultivated cottons have a high level of genetic variation in noncoding regions (Table 1), we suggest that at least some of these mutations have readjusted the CARE regulatory system of cultivated cotton following recurrent domestication. These changes probably increased the opportunities to produce beneficial agricultural traits in cotton.

### 4.3. Asymmetrical Selection Dynamics of the Subgenome in Cotton Domestication

As an allotetraploid species,* G. hirsutum *maintains its biparental subgenomes (A and D) in the nucleus. These two subgenomes provide double the genetic resources to cater to the requirements of domestication. Generally, within allopolyploid species, selection pressure is not always equal on both subgenomes. One of the two subgenomes may escape from parallel selection and accumulate diverse mutations. However, which subgenome would be selected for domestication traits depends on the species, and even the genes [40]. Asymmetrical selection dynamics for different subgenomes in polyploids are the rule rather than the exception [15, 25, 41, 42].

In upland cotton, we also found a bias in genetic variation between the A and D subgenomes (Figure 3). In terms of the total mutations, it seems reasonable that subgenome A possessed more SNP and InDels changes than subgenome D because subgenome A is larger [25]. However, it is worth noting that the dominant subgenome, which has more mutations, was not constant along the whole genome. Variation preference varied among different genetic positions and different chromosomes. For example, in the intergenic regions, the richest SNPs were found on chromosome 6 (384,769) in the A subgenome (Figure 3(c)). In contrast, in the upstream regions, the richest SNPs were detected on chromosome 5 (10,272) in the D subgenome (Figure 3(d)). Likewise, InDel mutations exhibited subgenomic asymmetry in the intergenic and upstream regions (Figure 3(d)). These results suggest that selective forces are independent of the two subgenomes.

## 5. Conclusion

The current study indicates that domestication has increased the complexity of the upland cotton genome, possibly through nonsynonymous substitutes, innovation of genes or transcripts by changing stop codons or shifting open reading frames in the coding regions, and reprogramming the regulatory system through CAREs in noncoding regions. Meanwhile, the subgenomes contributed differently during domestication. Subgenome A was more functionally conserved than subgenome D in the coding regions, and vice versa, subgenome D had more conserved sequences than subgenome A in the noncoding regions. However, because of limited sampling and lacking of complete knowledge on the directly primary ancestor of upland cotton in this study, we cannot strictly rule out the autapomorphies of current selected cotton accessions. The genetic divergences between wild and cultivated cottons might be amplified in such a study. Therefore, further deeper and wider studies are required to clarify the genetic mechanisms of upland cotton domestication.

## Figures and Tables

**Table 1:** Genetic divergence between *G. hirsutum *var. *yucatanense *and *G. hirsutum *acc. TM-1.

Category	Upstream	Exonic	Intronic	Downstream	Upstream/Downstream	Intergenic
SNP	149,469	132,271	283,859	123,958	7,742	5,931,601
InDel	43,047	3,952	51,547	32,258	2,066	589,730

Upstream: 1kb upstream region of a given gene; Downstream: 1kb downstream region of a given gene; Upstream/Downstream: 1kb upstream region of one gene, and meanwhile 1kb downstream region of another gene.

## Data Availability

The resequencing data used to support the findings of this study have been deposited in the GenBank under the accession No. SAMN07661378.

## Supplementary Materials

Supplementary 1 Table S1: nonsense SNP mutations between the cultivated and wild cottons.

Supplementary 2 Table S2: frame shift and nonsense indel mutations between the cultivated and wild cottons.

Supplementary 3 Table S3:* cis*-acting regulatory element variations in the upstream and downstream 2-kb regions of five genes between the cultivated and wild cottons.
