# Supplementary material for: Abundant Small Genetic Alterations after Upland Cotton Domestication
Source: Biomed Res Int. 2018 Dec 18;2018:9254302. doi: 10.1155/2018/9254302 (PMC6312614; doi:10.1155/2018/9254302)
Supplement: Supplementary 1 — Table S1: nonsense SNP mutations between the cultivated and wild cottons. [file 9254302.f1.pdf]

TABLE S1: Terminator mutations between the cultivated and wild cottons

Category	Gene	Exon	Nucleotide variation Cul /Position/ Wild	Protein variation Cul /Position/ Wild
Stop gain	Gh_A01G0066	7	C835T	Q279X
Stop gain	Gh_A01G0285	3	C1639T	Q547X
Stop gain	Gh_A01G0294	4	C1171T	Q391X
Stop gain	Gh_A01G0369	1	C131G	S44X
Stop gain	Gh_A01G0402	3	C427T	Q143X
Stop gain	Gh_A01G0585	1	C273A	Y91X
Stop loss	Gh_A01G0666	14	A2045C	X682S
Stop gain	Gh_A01G0675	8	C954A	Y318X
Stop gain	Gh_A01G0686	1	C34T	Q12X
Stop gain	Gh_A01G0709	1	T56A	L19X
Stop loss	Gh_A01G0776	3	T343C	X115R
Stop gain	Gh_A01G0841	1	G1042T	E348X
Stop gain	Gh_A01G0946	5	G1282T	E428X
Stop gain	Gh_A01G0947	1	C40T	Q14X
Stop gain	Gh_A01G0948	7	A1036T	K346X
Stop gain	Gh_A01G1036	5	C486A	Y162X
Stop loss	Gh_A01G1039	1	A624C	X208C
Stop gain	Gh_A01G1172	9	C1717T	R573X
Stop gain	Gh_A01G1224	2	G647A	W216X
Stop gain	Gh_A01G1301	1	G173A	W58X
Stop gain	Gh_A01G1301	1	C916T	Q306X
Stop gain	Gh_A01G1301	1	C1066T	Q356X
Stop gain	Gh_A01G1301	1	C1333T	Q445X
Stop gain	Gh_A01G1343	1	A79T	K27X
Stop gain	Gh_A01G1666	15	C1825T	Q609X
Stop loss	Gh_A01G1738	3	T181G	X61E
Stop loss	Gh_A01G1849	16	T2128A	X710R
Stop loss	Gh_A01G1919	9	T1207C	X403Q
Stop gain	Gh_A02G0098	3	C2931G	Y977X
Stop gain	Gh_A02G0105	11	C4347G	Y1449X
Stop gain	Gh_A02G0107	1	G951A	W317X
Stop loss	Gh_A02G0107	7	T1906C	X636R
Stop gain	Gh_A02G0124	2	G760T	E254X
Stop gain	Gh_A02G0147	5	C712T	R238X
Stop gain	Gh_A02G0258	1	T114A	Y38X
Stop gain	Gh_A02G0259	1	G97T	E33X
Stop gain	Gh_A02G0261	3	C601T	R201X
Stop loss	Gh_A02G0314	1	T562A	X188K
Stop gain	Gh_A02G0315	1	G46T	E16X
Stop gain	Gh_A02G0384	2	C434G	S145X
Stop gain	Gh_A02G0401	5	C1989A	C663X
Stop gain	Gh_A02G0409	13	G1467A	W489X
Stop loss	Gh_A02G0476	1	T181C	X61Q
Stop loss	Gh_A02G0604	2	A261G	X87W
Stop gain	Gh_A02G0724	1	T584A	L195X

Stop loss	Gh_A02G0725	1	T1042A	X348K
Stop gain	Gh_A02G0803	3	G1171T	E391X
Stop loss	Gh_A02G1283	2	T199C	X67Q
Stop loss	Gh_A02G1509	2	T1234G	X412E
Stop gain	Gh_A02G1558	1	G47A	W16X
Stop gain	Gh_A02G1560	2	G112T	E38X
Stop gain	Gh_A03G0050	4	T782G	L261X
Stop gain	Gh_A03G0058	4	G523T	G175X
Stop gain	Gh_A03G0061	7	G1680A	W560X
Stop loss	Gh_A03G0150	1	T175C	X59R
Stop gain	Gh_A03G0289	1	C868T	Q290X
Stop loss	Gh_A03G0326	7	A461T	X154L
Stop gain	Gh_A03G0522	2	C933A	C311X
Stop gain	Gh_A03G0571	3	T660A	C220X
Stop loss	Gh_A03G0608	2	A425G	X142W
Stop gain	Gh_A03G0618	3	T686A	L229X
Stop loss	Gh_A03G0706	9	A872T	X291L
Stop gain	Gh_A03G0725	1	G463T	E155X
Stop gain	Gh_A03G0746	1	G182A	W61X
Stop gain	Gh_A03G0751	2	C1441T	Q481X
Stop gain	Gh_A03G0762	3	C493T	Q165X
Stop gain	Gh_A03G0869	1	C68A	S23X
Stop gain	Gh_A03G0945	4	G439T	E147X
Stop gain	Gh_A03G0966	1	C256T	Q86X
Stop gain	Gh_A03G0967	1	C541T	Q181X
Stop gain	Gh_A03G1049	1	T1301A	L434X
Stop gain	Gh_A03G1077	1	G433T	E145X
Stop gain	Gh_A03G1126	7	C3874T	Q1292X
Stop gain	Gh_A03G1249	1	C175T	Q59X
Stop gain	Gh_A03G1270	5	G622T	E208X
Stop loss	Gh_A03G1276	1	T472C	X158R
Stop gain	Gh_A03G1298	1	C434G	S145X
Stop loss	Gh_A03G1301	7	T505C	X169Q
Stop gain	Gh_A03G1381	1	C29G	S10X
Stop loss	Gh_A03G1396	1	T853G	X285E
Stop loss	Gh_A03G1421	7	A1082C	X361S
Stop gain	Gh_A03G1467	3	G689A	W230X
Stop gain	Gh_A03G1468	3	G334T	E112X
Stop loss	Gh_A03G1610	1	T808G	X270E
Stop loss	Gh_A03G1636	1	T682C	X228Q
Stop gain	Gh_A04G0123	1	T1088A	L363X
Stop gain	Gh_A04G0154	15	C3415T	Q1139X
Stop gain	Gh_A04G0165	2	C1567T	R523X
Stop gain	Gh_A04G0174	2	C261G	Y87X
Stop gain	Gh_A04G0183	4	G2161T	E721X
Stop gain	Gh_A04G0186	5	C634T	Q212X
Stop gain	Gh_A04G0203	7	T2229A	C743X
Stop gain	Gh_A04G0242	2	C257A	S86X

Stop loss	Gh_A04G0260	1	A978C	X326Y
Stop gain	Gh_A04G0264	1	T309G	Y103X
Stop gain	Gh_A04G0284	2	G721T	E241X
Stop gain	Gh_A04G0427	4	C289T	Q97X
Stop loss	Gh_A04G0801	1	T2026A	X676K
Stop gain	Gh_A04G1020	1	G185A	W62X
Stop loss	Gh_A04G1067	1	T712C	X238R
Stop gain	Gh_A04G1072	8	C1663T	Q555X
Stop gain	Gh_A04G1131	1	C435A	C145X
Stop gain	Gh_A05G0090	1	C298T	R100X
Stop loss	Gh_A05G0297	1	A794G	X265W
Stop gain	Gh_A05G0371	4	C495A	C165X
Stop gain	Gh_A05G0854	4	C642A	C214X
Stop loss	Gh_A05G0943	2	A468T	X156Y
Stop gain	Gh_A05G1375	2	C679T	Q227X
Stop gain	Gh_A05G1479	2	C602A	S201X
Stop loss	Gh_A05G1777	1	A266C	X89S
Stop gain	Gh_A05G1938	2	C691T	R231X
Stop gain	Gh_A05G1947	2	T189G	Y63X
Stop loss	Gh_A05G2333	2	T2209C	X737R
Stop gain	Gh_A05G2610	2	C1960T	R654X
Stop loss	Gh_A05G2725	4	T349C	X117R
Stop loss	Gh_A05G3075	1	T1444C	X482Q
Stop gain	Gh_A05G3143	2	A1009T	R337X
Stop loss	Gh_A05G3145	1	T586C	X196Q
Stop loss	Gh_A05G3259	2	G693T	X231Y
Stop loss	Gh_A05G3509	1	A1493T	X498L
Stop loss	Gh_A05G3602	1	A365T	X122L
Stop loss	Gh_A06G0038	3	G1350C	X450Y
Stop gain	Gh_A06G0107	3	T1394A	L465X
Stop gain	Gh_A06G0108	1	G173A	W58X
Stop loss	Gh_A06G0122	1	G510C	X170Y
Stop gain	Gh_A06G0167	1	C734G	S245X
Stop loss	Gh_A06G0198	2	T1303G	X435G
Stop gain	Gh_A06G0291	1	C1781G	S594X
Stop gain	Gh_A06G0306	2	C442T	R148X
Stop loss	Gh_A06G0352	1	T169G	X57E
Stop gain	Gh_A06G0372	1	G84A	W28X
Stop gain	Gh_A06G0372	1	C508T	R170X
Stop gain	Gh_A06G0372	1	T770A	L257X
Stop gain	Gh_A06G0387	4	G343T	E115X
Stop gain	Gh_A06G0389	7	C871T	Q291X
Stop loss	Gh_A06G0416	1	A191G	X64W
Stop gain	Gh_A06G0441	2	G107A	W36X
Stop gain	Gh_A06G0579	4	G1182A	W394X
Stop gain	Gh_A06G0630	2	C1024T	Q342X
Stop gain	Gh_A06G0690	4	G274T	E92X
Stop loss	Gh_A06G0730	4	G1034C	X345S

Stop gain	Gh_A06G0908	3	C91T	R31X
Stop gain	Gh_A06G0938	1	G1152A	W384X
Stop gain	Gh_A06G0942	6	T654A	Y218X
Stop gain	Gh_A06G1078	1	A271T	K91X
Stop gain	Gh_A06G1504	4	G466T	E156X
Stop gain	Gh_A06G1508	2	C2383T	R795X
Stop gain	Gh_A06G1583	8	T1625A	L542X
Stop gain	Gh_A06G1656	2	C2185T	R729X
Stop loss	Gh_A06G1701	3	A927C	X309Y
Stop gain	Gh_A06G1714	2	T42G	Y14X
Stop gain	Gh_A06G1769	3	G1093T	G365X
Stop gain	Gh_A06G1776	1	C142T	Q48X
Stop gain	Gh_A06G1804	2	C178T	Q60X
Stop gain	Gh_A07G0134	1	G653A	W218X
Stop gain	Gh_A07G0196	5	A895T	R299X
Stop gain	Gh_A07G0314	1	C976T	Q326X
Stop gain	Gh_A07G0322	2	C24A	Y8X
Stop gain	Gh_A07G0401	2	G287A	W96X
Stop gain	Gh_A07G0406	2	C226T	R76X
Stop loss	Gh_A07G0448	1	T2272C	X758Q
Stop gain	Gh_A07G0476	3	C595T	R199X
Stop gain	Gh_A07G0491	3	C928T	R310X
Stop gain	Gh_A07G0491	2	C637T	R213X
Stop gain	Gh_A07G0597	2	G610T	G204X
Stop gain	Gh_A07G0693	1	C379T	Q127X
Stop gain	Gh_A07G0693	2	C844T	Q282X
Stop gain	Gh_A07G0704	1	G80A	W27X
Stop gain	Gh_A07G0727	1	C256T	Q86X
Stop gain	Gh_A07G0727	1	G263A	W88X
Stop loss	Gh_A07G0778	8	T1462C	X488R
Stop gain	Gh_A07G0782	1	C115T	R39X
Stop gain	Gh_A07G0908	3	C402A	Y134X
Stop gain	Gh_A07G1322	3	C1168T	Q390X
Stop gain	Gh_A07G1322	12	C2029T	Q677X
Stop loss	Gh_A07G1446	2	A2901T	X967C
Stop gain	Gh_A07G1446	2	C2107T	R703X
Stop loss	Gh_A07G1456	1	T298G	X100E
Stop gain	Gh_A07G1503	5	C913T	R305X
Stop gain	Gh_A07G1542	4	C1033T	Q345X
Stop gain	Gh_A07G1634	1	C328T	Q110X
Stop gain	Gh_A07G1647	1	C1201T	R401X
Stop loss	Gh_A07G1648	1	T1897G	X633E
Stop loss	Gh_A07G1649	1	A939T	X313C
Stop gain	Gh_A07G1694	2	G167A	W56X
Stop gain	Gh_A07G1707	3	G172T	E58X
Stop gain	Gh_A07G1742	2	T653G	L218X
Stop gain	Gh_A07G1756	8	G1201T	G401X
Stop gain	Gh_A07G1832	1	A88T	K30X

Stop gain	Gh_A07G1837	4	T1401A	Y467X
Stop loss	Gh_A07G1867	1	T814C	X272Q
Stop gain	Gh_A07G1976	4	G1396T	G466X
Stop gain	Gh_A07G2036	1	C136T	R46X
Stop gain	Gh_A08G0185	3	C1729T	R577X
Stop gain	Gh_A08G0396	5	T464A	L155X
Stop loss	Gh_A08G0413	1	G2222C	X741S
Stop gain	Gh_A08G0522	3	C945A	Y315X
Stop gain	Gh_A08G0708	1	C535T	R179X
Stop gain	Gh_A08G0713	2	C679T	Q227X
Stop gain	Gh_A08G0718	1	C229T	Q77X
Stop loss	Gh_A08G0974	8	T1309C	X437Q
Stop gain	Gh_A08G0991	9	A1057T	K353X
Stop gain	Gh_A08G1193	1	G67T	E23X
Stop loss	Gh_A08G1237	12	T1618A	X540K
Stop gain	Gh_A08G1499	2	G151T	E51X
Stop loss	Gh_A08G1636	6	T1306C	X436R
Stop loss	Gh_A08G1847	1	T1033C	X345R
Stop gain	Gh_A08G1886	1	A544T	R182X
Stop gain	Gh_A08G1943	2	G241T	G81X
Stop gain	Gh_A08G1989	16	C4748A	S1583X
Stop loss	Gh_A08G2067	3	T862C	X288Q
Stop loss	Gh_A08G2153	3	T862C	X288Q
Stop gain	Gh_A08G2164	5	A973T	K325X
Stop gain	Gh_A08G2262	1	G303A	W101X
Stop loss	Gh_A08G2276	6	T910C	X304Q
Stop loss	Gh_A08G2276	6	A911C	X304S
Stop gain	Gh_A09G0099	1	T735G	Y245X
Stop gain	Gh_A09G0131	1	A151T	K51X
Stop gain	Gh_A09G0147	1	C42G	Y14X
Stop gain	Gh_A09G0151	3	C1313A	S438X
Stop loss	Gh_A09G0181	3	G1835C	X612S
Stop gain	Gh_A09G0228	1	G726A	W242X
Stop gain	Gh_A09G0228	1	C1450T	Q484X
Stop gain	Gh_A09G0267	1	G88T	G30X
Stop loss	Gh_A09G0313	2	T493C	X165R
Stop loss	Gh_A09G0370	4	A1031C	X344S
Stop gain	Gh_A09G0444	2	T812A	L271X
Stop gain	Gh_A09G0531	3	C508T	Q170X
Stop gain	Gh_A09G0648	3	G615A	W205X
Stop gain	Gh_A09G0834	24	C2639G	S880X
Stop gain	Gh_A09G0902	5	C1291T	Q431X
Stop loss	Gh_A09G1073	3	A1478T	X493L
Stop gain	Gh_A09G1127	2	C516A	Y172X
Stop gain	Gh_A09G1186	5	T1277A	L426X
Stop loss	Gh_A09G1205	7	T2728G	X910G
Stop gain	Gh_A09G1300	1	C364T	Q122X
Stop gain	Gh_A09G1308	4	C335A	S112X

Stop gain	Gh_A09G1432	1	C210A	Y70X
Stop gain	Gh_A09G1454	3	G458A	W153X
Stop gain	Gh_A09G1489	2	G519A	W173X
Stop gain	Gh_A10G0126	3	G1426T	E476X
Stop gain	Gh_A10G0206	3	A388T	R130X
Stop loss	Gh_A10G0503	4	T466C	X156R
Stop gain	Gh_A10G0526	2	C610T	Q204X
Stop gain	Gh_A10G0965	1	C1420T	Q474X
Stop gain	Gh_A10G0974	2	C254A	S85X
Stop loss	Gh_A10G1004	1	A827T	X276L
Stop gain	Gh_A10G1054	1	C95A	S32X
Stop gain	Gh_A10G1130	4	G3571T	G1191X
Stop gain	Gh_A10G1130	2	G1570T	E524X
Stop gain	Gh_A10G1133	6	C865T	R289X
Stop gain	Gh_A10G1159	6	C1384T	R462X
Stop loss	Gh_A10G1181	1	T367G	X123E
Stop loss	Gh_A10G1374	3	T499G	X167G
Stop gain	Gh_A10G1438	7	G693A	W231X
Stop gain	Gh_A10G1569	1	G193T	E65X
Stop loss	Gh_A10G1569	1	T700C	X234Q
Stop gain	Gh_A10G1635	5	C1639T	Q547X
Stop gain	Gh_A10G1780	2	C310T	R104X
Stop gain	Gh_A10G1837	14	C1404G	Y468X
Stop gain	Gh_A10G1865	4	G532T	E178X
Stop gain	Gh_A10G1906	1	C1372T	Q458X
Stop gain	Gh_A10G2073	5	C1787A	S596X
Stop gain	Gh_A10G2103	1	C641G	S214X
Stop gain	Gh_A10G2148	2	C232T	Q78X
Stop loss	Gh_A11G0099	8	A1226G	X409W
Stop gain	Gh_A11G0418	1	T42A	C14X
Stop gain	Gh_A11G0437	5	G371A	W124X
Stop loss	Gh_A11G0737	5	G482T	X161L
Stop loss	Gh_A11G0864	6	T895C	X299Q
Stop gain	Gh_A11G0877	1	G1297T	E433X
Stop gain	Gh_A11G0942	1	C370T	R124X
Stop loss	Gh_A11G1341	1	T331G	X111E
Stop loss	Gh_A11G1363	2	T715C	X239R
Stop gain	Gh_A11G1363	2	G310T	E104X
Stop gain	Gh_A11G1401	1	C992A	S331X
Stop gain	Gh_A11G1414	7	G805T	E269X
Stop loss	Gh_A11G1534	1	A1128T	X376Y
Stop gain	Gh_A11G1559	4	C394T	R132X
Stop loss	Gh_A11G1777	10	T1441C	X481Q
Stop gain	Gh_A11G1854	1	G67T	E23X
Stop gain	Gh_A11G1877	2	C1359A	C453X
Stop gain	Gh_A11G1896	3	C928T	Q310X
Stop gain	Gh_A11G1902	1	C1630T	R544X
Stop gain	Gh_A11G2009	3	G2066A	W689X

Stop gain	Gh_A11G2099	1	C40T	Q14X
Stop gain	Gh_A11G2115	4	G347A	W116X
Stop gain	Gh_A11G2150	1	C997T	R333X
Stop gain	Gh_A11G2184	1	G433T	E145X
Stop loss	Gh_A11G2249	2	A293T	X98L
Stop gain	Gh_A11G2328	1	T60G	Y20X
Stop gain	Gh_A11G2503	3	G154T	E52X
Stop gain	Gh_A11G2505	8	G3004T	E1002X
Stop loss	Gh_A11G2512	4	G372T	X124Y
Stop gain	Gh_A11G2680	12	G4390T	G1464X
Stop loss	Gh_A11G2752	2	T685C	X229Q
Stop loss	Gh_A11G2831	1	T565G	X189E
Stop gain	Gh_A11G2949	6	G985T	G329X
Stop gain	Gh_A12G0008	7	G2286A	W762X
Stop gain	Gh_A12G0083	2	C547T	Q183X
Stop gain	Gh_A12G0383	1	G259T	E87X
Stop gain	Gh_A12G0492	2	C151T	R51X
Stop gain	Gh_A12G0500	1	G17A	W6X
Stop loss	Gh_A12G0662	1	T505C	X169Q
Stop gain	Gh_A12G0731	6	T369A	Y123X
Stop gain	Gh_A12G0731	8	C457T	Q153X
Stop gain	Gh_A12G0739	11	C1701G	Y567X
Stop loss	Gh_A12G0748	2	T937A	X313K
Stop gain	Gh_A12G0765	1	C40T	Q14X
Stop gain	Gh_A12G0825	11	G836A	W279X
Stop gain	Gh_A12G0834	2	C73T	Q25X
Stop gain	Gh_A12G0847	2	C220T	Q74X
Stop gain	Gh_A12G0884	2	C135A	C45X
Stop loss	Gh_A12G0905	2	T1474C	X492Q
Stop gain	Gh_A12G0907	2	G561A	W187X
Stop gain	Gh_A12G0908	2	C356G	S119X
Stop gain	Gh_A12G0926	6	C763T	R255X
Stop loss	Gh_A12G0994	1	T472C	X158R
Stop gain	Gh_A12G1019	2	G625T	E209X
Stop gain	Gh_A12G1023	3	C1653A	C551X
Stop gain	Gh_A12G1023	3	G1397A	W466X
Stop loss	Gh_A12G1026	2	T1249G	X417E
Stop gain	Gh_A12G1036	2	C667T	R223X
Stop gain	Gh_A12G1179	1	A418T	K140X
Stop gain	Gh_A12G1464	4	C719A	S240X
Stop loss	Gh_A12G1466	2	T1489G	X497E
Stop loss	Gh_A12G1540	8	A833C	X278S
Stop gain	Gh_A12G1825	1	C94T	Q32X
Stop gain	Gh_A12G1846	5	T1094A	L365X
Stop gain	Gh_A12G2089	2	G320A	W107X
Stop gain	Gh_A12G2126	1	G661T	E221X
Stop gain	Gh_A12G2140	2	T282A	Y94X
Stop gain	Gh_A13G0055	2	A1726T	R576X

Stop gain	Gh_A13G0174	1	G364T	G122X
Stop gain	Gh_A13G0176	2	C321A	Y107X
Stop loss	Gh_A13G0209	1	A483T	X161Y
Stop loss	Gh_A13G0870	3	T1021C	X341R
Stop gain	Gh_A13G0875	6	C592T	Q198X
Stop loss	Gh_A13G0911	2	T235A	X79K
Stop loss	Gh_A13G0965	11	A1562T	X521L
Stop loss	Gh_A13G0975	3	T1570C	X524Q
Stop gain	Gh_A13G0980	1	G646T	G216X
Stop gain	Gh_A13G0980	1	C103T	Q35X
Stop loss	Gh_A13G1004	8	G714T	X238Y
Stop gain	Gh_A13G1135	2	C635A	S212X
Stop gain	Gh_A13G1135	9	C1924T	R642X
Stop gain	Gh_A13G1234	3	A223T	R75X
Stop gain	Gh_A13G1234	6	C1105T	Q369X
Stop loss	Gh_A13G1237	2	A524G	X175W
Stop gain	Gh_A13G1690	8	C763T	R255X
Stop gain	Gh_A13G1718	1	C172T	Q58X
Stop gain	Gh_A13G1756	2	A1168T	K390X
Stop gain	Gh_A13G1766	2	A379T	K127X
Stop gain	Gh_A13G2027	3	T473A	L158X
Stop gain	Gh_A13G2049	2	C577T	Q193X
Stop gain	Gh_A13G2064	5	C228A	C76X
Stop loss	Gh_D01G0138	11	A1472C	X491S
Stop gain	Gh_D01G0144	3	G454T	E152X
Stop loss	Gh_D01G0267	2	T1771C	X591Q
Stop gain	Gh_D01G0334	2	A280T	K94X
Stop gain	Gh_D01G0349	2	C1542A	Y514X
Stop gain	Gh_D01G0361	4	C1678T	Q560X
Stop loss	Gh_D01G0362	3	T1321C	X441R
Stop gain	Gh_D01G0365	21	T2418A	Y806X
Stop loss	Gh_D01G0477	1	T1249C	X417R
Stop gain	Gh_D01G0558	4	C202T	Q68X
Stop loss	Gh_D01G0755	6	G854C	X285S
Stop gain	Gh_D01G0918	8	C745T	R249X
Stop gain	Gh_D01G1016	2	C475T	R159X
Stop gain	Gh_D01G1031	4	C370T	R124X
Stop loss	Gh_D01G1093	4	T391C	X131Q
Stop gain	Gh_D01G1167	1	G86A	W29X
Stop gain	Gh_D01G1282	5	C880T	R294X
Stop gain	Gh_D01G1305	1	G281A	W94X
Stop gain	Gh_D01G1307	1	G316T	G106X
Stop gain	Gh_D01G1326	8	C643T	Q215X
Stop loss	Gh_D01G1398	2	A348G	X116W
Stop gain	Gh_D01G1409	2	G494A	W165X
Stop gain	Gh_D01G1417	3	C373T	Q125X
Stop gain	Gh_D01G1422	2	C118T	Q40X
Stop gain	Gh_D01G1425	1	C38G	S13X

Stop loss	Gh_D01G1442	1	T349C	X117R
Stop gain	Gh_D01G1442	1	C82T	Q28X
Stop gain	Gh_D01G1445	3	C799T	Q267X
Stop gain	Gh_D01G1454	1	G77A	W26X
Stop loss	Gh_D01G1456	1	T577C	X193Q
Stop gain	Gh_D01G1494	1	C325T	R109X
Stop gain	Gh_D01G1538	6	C670T	Q224X
Stop gain	Gh_D01G1614	1	G182A	W61X
Stop gain	Gh_D01G1624	1	A109T	K37X
Stop gain	Gh_D01G1767	2	C193T	Q65X
Stop gain	Gh_D01G2202	18	C2059T	R687X
Stop gain	Gh_D02G0046	1	C576G	Y192X
Stop loss	Gh_D02G0094	5	T1876G	X626G
Stop gain	Gh_D02G0106	1	G1647A	W549X
Stop gain	Gh_D02G0106	1	C552A	Y184X
Stop gain	Gh_D02G0119	5	C397T	R133X
Stop gain	Gh_D02G0127	8	C2726A	S909X
Stop gain	Gh_D02G0137	2	C413A	S138X
Stop gain	Gh_D02G0162	1	G1573T	E525X
Stop gain	Gh_D02G0162	1	G1934A	W645X
Stop gain	Gh_D02G0172	2	T1488A	C496X
Stop loss	Gh_D02G0179	1	T718G	X240E
Stop gain	Gh_D02G0193	1	C7T	R3X
Stop gain	Gh_D02G0202	6	C2444A	S815X
Stop gain	Gh_D02G0218	1	C1345T	Q449X
Stop gain	Gh_D02G0218	1	A922T	K308X
Stop gain	Gh_D02G0257	2	T1839A	Y613X
Stop loss	Gh_D02G0277	4	A1841G	X614W
Stop gain	Gh_D02G0398	3	G124T	G42X
Stop gain	Gh_D02G0424	1	C55T	Q19X
Stop gain	Gh_D02G0451	1	G278A	W93X
Stop gain	Gh_D02G0521	1	C310T	R104X
Stop gain	Gh_D02G0766	1	G249A	W83X
Stop gain	Gh_D02G0832	1	C836G	S279X
Stop gain	Gh_D02G0833	1	G181T	G61X
Stop gain	Gh_D02G0907	2	C824G	S275X
Stop gain	Gh_D02G0916	1	T300A	C100X
Stop gain	Gh_D02G0942	4	G1861T	G621X
Stop gain	Gh_D02G1042	1	T74A	L25X
Stop gain	Gh_D02G1094	3	C133T	Q45X
Stop gain	Gh_D02G1418	2	T710A	L237X
Stop gain	Gh_D02G1440	1	C83A	S28X
Stop gain	Gh_D02G1470	2	A472T	R158X
Stop loss	Gh_D02G1472	1	A1154T	X385L
Stop gain	Gh_D02G1872	1	T194A	L65X
Stop loss	Gh_D02G2086	1	G923T	X308L
Stop gain	Gh_D02G2146	2	C423G	Y141X
Stop gain	Gh_D02G2252	13	A1732T	R578X

Stop gain	Gh_D02G2260	6	T770A	L257X
Stop gain	Gh_D02G2260	4	G628T	G210X
Stop gain	Gh_D02G2276	1	C373T	Q125X
Stop gain	Gh_D02G2347	2	A1495T	K499X
Stop gain	Gh_D03G0507	1	G762A	W254X
Stop gain	Gh_D03G0724	1	C208T	Q70X
Stop gain	Gh_D03G0807	3	C757T	Q253X
Stop gain	Gh_D03G0875	7	C541T	Q181X
Stop gain	Gh_D03G1129	4	C595T	Q199X
Stop gain	Gh_D03G1291	3	G534A	W178X
Stop gain	Gh_D03G1303	10	C1018T	R340X
Stop gain	Gh_D03G1365	2	C217T	Q73X
Stop loss	Gh_D03G1448	2	A293T	X98L
Stop gain	Gh_D03G1527	4	C5398T	Q1800X
Stop gain	Gh_D03G1527	4	C6494A	S2165X
Stop gain	Gh_D03G1527	4	A7609T	K2537X
Stop gain	Gh_D04G0011	1	G71A	W24X
Stop gain	Gh_D04G0046	1	G764A	W255X
Stop gain	Gh_D04G0175	1	T1107A	Y369X
Stop gain	Gh_D04G0202	2	G601T	G201X
Stop gain	Gh_D04G0250	1	G225A	W75X
Stop gain	Gh_D04G0388	3	C184T	R62X
Stop loss	Gh_D04G0434	1	T337C	X113Q
Stop gain	Gh_D04G0471	2	G620A	W207X
Stop gain	Gh_D04G0474	2	G513A	W171X
Stop gain	Gh_D04G0499	4	C494G	S165X
Stop gain	Gh_D04G0592	2	C418T	Q140X
Stop gain	Gh_D04G0613	1	C44A	S15X
Stop gain	Gh_D04G0629	2	C934T	Q312X
Stop gain	Gh_D04G0647	9	C812G	S271X
Stop gain	Gh_D04G0650	2	C304T	Q102X
Stop gain	Gh_D04G0653	5	C925T	Q309X
Stop loss	Gh_D04G0769	1	T268C	X90Q
Stop gain	Gh_D04G0898	1	C403T	R135X
Stop gain	Gh_D04G0905	6	A898T	R300X
Stop loss	Gh_D04G0912	2	T682C	X228Q
Stop loss	Gh_D04G0954	1	T163C	X55Q
Stop gain	Gh_D04G1194	3	C1828T	Q610X
Stop gain	Gh_D04G1201	1	C1429T	Q477X
Stop gain	Gh_D04G1201	1	G1265A	W422X
Stop gain	Gh_D04G1216	2	C749A	S250X
Stop loss	Gh_D04G1222	2	T928G	X310G
Stop gain	Gh_D04G1290	4	T230A	L77X
Stop loss	Gh_D04G1351	1	T190C	X64Q
Stop gain	Gh_D04G1394	2	C456G	Y152X
Stop gain	Gh_D04G1429	1	A658T	K220X
Stop loss	Gh_D04G1440	7	A1530G	X510W
Stop loss	Gh_D04G1566	2	T547C	X183R

Stop loss	Gh_D04G1684	1	T244C	X82Q
Stop gain	Gh_D04G1725	2	A571T	R191X
Stop gain	Gh_D04G1763	3	C886T	R296X
Stop gain	Gh_D05G0428	2	G421T	E141X
Stop loss	Gh_D05G0547	7	T2215A	X739R
Stop gain	Gh_D05G0712	3	T351G	Y117X
Stop loss	Gh_D05G1131	21	G2201T	X734L
Stop gain	Gh_D05G1136	5	C1116A	Y372X
Stop gain	Gh_D05G1160	1	G72A	W24X
Stop gain	Gh_D05G1166	1	G305A	W102X
Stop gain	Gh_D05G1169	6	C559T	Q187X
Stop gain	Gh_D05G1233	1	G132A	W44X
Stop loss	Gh_D05G1249	2	T187C	X63Q
Stop gain	Gh_D05G1277	2	C256T	R86X
Stop gain	Gh_D05G1556	1	G172T	E58X
Stop gain	Gh_D05G1622	1	G16T	E6X
Stop gain	Gh_D05G1640	1	C79T	R27X
Stop gain	Gh_D05G1945	2	A448T	K150X
Stop gain	Gh_D05G1976	1	C112T	R38X
Stop gain	Gh_D05G1982	1	T201A	C67X
Stop loss	Gh_D05G1999	2	T910G	X304E
Stop gain	Gh_D05G2156	6	C742T	R248X
Stop loss	Gh_D05G2288	1	T883G	X295E
Stop loss	Gh_D05G2646	4	T1012C	X338Q
Stop loss	Gh_D05G2852	1	T556C	X186Q
Stop loss	Gh_D05G3264	5	T979C	X327Q
Stop gain	Gh_D05G3428	1	G1213T	G405X
Stop gain	Gh_D05G3519	1	G333A	W111X
Stop loss	Gh_D05G3574	1	T454C	X152Q
Stop gain	Gh_D05G3579	1	G698A	W233X
Stop gain	Gh_D05G3615	1	C748T	Q250X
Stop gain	Gh_D05G3627	1	C57A	C19X
Stop gain	Gh_D05G3653	10	A1726T	K576X
Stop gain	Gh_D05G3667	11	G3287A	W1096X
Stop gain	Gh_D05G3667	2	G164A	W55X
Stop gain	Gh_D06G0026	3	C642A	C214X
Stop gain	Gh_D06G0306	1	G42A	W14X
Stop gain	Gh_D06G0314	4	C442T	Q148X
Stop gain	Gh_D06G0338	1	A226T	K76X
Stop gain	Gh_D06G0342	1	C76T	Q26X
Stop gain	Gh_D06G0473	1	A163T	K55X
Stop gain	Gh_D06G0533	6	G362A	W121X
Stop gain	Gh_D06G0549	2	G66A	W22X
Stop gain	Gh_D06G0558	1	T20A	L7X
Stop gain	Gh_D06G0634	1	T77A	L26X
Stop gain	Gh_D06G0688	2	C472T	Q158X
Stop gain	Gh_D06G0692	10	G2041T	E681X
Stop gain	Gh_D06G0696	1	C343T	Q115X

Stop loss	Gh_D06G0707	1	A362G	X121W
Stop gain	Gh_D06G0714	1	C1213T	Q405X
Stop gain	Gh_D06G0736	2	G200A	W67X
Stop gain	Gh_D06G0787	4	A700T	K234X
Stop gain	Gh_D06G0812	1	C498A	Y166X
Stop gain	Gh_D06G0834	1	G304T	E102X
Stop gain	Gh_D06G0842	4	A676T	K226X
Stop gain	Gh_D06G0850	1	C49T	Q17X
Stop gain	Gh_D06G0852	1	C868T	R290X
Stop gain	Gh_D06G0892	2	C217T	R73X
Stop gain	Gh_D06G0973	2	C226T	Q76X
Stop gain	Gh_D06G0989	12	T1053G	Y351X
Stop gain	Gh_D06G0995	3	C258G	Y86X
Stop gain	Gh_D06G1062	6	A502T	K168X
Stop loss	Gh_D06G1116	2	T172A	X58K
Stop gain	Gh_D06G1263	1	C97T	Q33X
Stop gain	Gh_D06G1271	3	G884A	W295X
Stop gain	Gh_D06G1319	4	A253T	K85X
Stop gain	Gh_D06G1331	1	C213A	C71X
Stop loss	Gh_D06G1331	2	T1318C	X440R
Stop loss	Gh_D06G1333	2	T703C	X235R
Stop gain	Gh_D06G1350	4	G1541A	W514X
Stop loss	Gh_D06G1435	1	T1285C	X429R
Stop gain	Gh_D06G1557	3	A178T	R60X
Stop gain	Gh_D06G1606	2	T1664A	L555X
Stop gain	Gh_D06G1611	2	G317A	W106X
Stop gain	Gh_D06G1639	1	C16T	Q6X
Stop gain	Gh_D06G1643	2	C58T	R20X
Stop gain	Gh_D06G1699	1	G56A	W19X
Stop gain	Gh_D06G1871	2	T191A	L64X
Stop gain	Gh_D06G1880	1	G67T	E23X
Stop loss	Gh_D06G1908	3	T643G	X215E
Stop loss	Gh_D06G2028	1	T322C	X108Q
Stop loss	Gh_D06G2070	1	T832C	X278Q
Stop loss	Gh_D06G2102	3	A632G	X211W
Stop gain	Gh_D06G2157	1	T1083A	C361X
Stop loss	Gh_D06G2174	8	T1705C	X569R
Stop loss	Gh_D07G0210	11	T2245C	X749Q
Stop gain	Gh_D07G0776	4	G1999T	E667X
Stop gain	Gh_D07G0871	9	G913T	E305X
Stop loss	Gh_D07G0874	1	A3057C	X1019Y
Stop gain	Gh_D07G0909	6	C460T	Q154X
Stop gain	Gh_D07G1111	3	T509A	L170X
Stop loss	Gh_D07G1337	1	G762T	X254Y
Stop loss	Gh_D07G1768	5	G728T	X243L
Stop gain	Gh_D07G1953	1	C1744T	Q582X
Stop gain	Gh_D07G1983	5	T2340A	Y780X
Stop gain	Gh_D07G1998	2	G505T	G169X

Stop gain	Gh_D07G2112	1	G164A	W55X
Stop gain	Gh_D07G2112	1	G165A	W55X
Stop gain	Gh_D07G2122	4	C238T	Q80X
Stop gain	Gh_D07G2130	5	C376T	Q126X
Stop loss	Gh_D07G2134	6	G1368C	X456Y
Stop gain	Gh_D07G2191	7	G1108T	E370X
Stop gain	Gh_D07G2249	1	C175T	R59X
Stop gain	Gh_D07G2347	4	G584A	W195X
Stop gain	Gh_D07G2352	1	C373T	Q125X
Stop gain	Gh_D08G0280	8	A976T	K326X
Stop gain	Gh_D08G0885	2	G255A	W85X
Stop loss	Gh_D08G0932	1	T445G	X149E
Stop gain	Gh_D08G1131	1	C20A	S7X
Stop gain	Gh_D08G1163	1	G131A	W44X
Stop loss	Gh_D08G1208	4	T271C	X91Q
Stop gain	Gh_D08G1214	1	C943T	Q315X
Stop gain	Gh_D08G1650	8	A994T	K332X
Stop gain	Gh_D08G1786	1	C328T	Q110X
Stop loss	Gh_D08G1995	11	T2023G	X675G
Stop gain	Gh_D08G2092	1	C55T	Q19X
Stop gain	Gh_D08G2124	1	C643T	R215X
Stop gain	Gh_D08G2238	3	T1017G	Y339X
Stop gain	Gh_D08G2244	1	C187T	Q63X
Stop gain	Gh_D08G2318	1	A1567T	R523X
Stop gain	Gh_D09G0029	24	G2767T	E923X
Stop gain	Gh_D09G0056	4	T788A	L263X
Stop gain	Gh_D09G0077	1	G66A	W22X
Stop gain	Gh_D09G0184	1	C1131A	Y377X
Stop loss	Gh_D09G0397	1	T208C	X70Q
Stop loss	Gh_D09G0458	1	T169C	X57R
Stop gain	Gh_D09G0706	1	G328T	E110X
Stop gain	Gh_D09G0863	8	A1129T	K377X
Stop gain	Gh_D09G0900	4	C535T	Q179X
Stop gain	Gh_D09G1056	2	T767G	L256X
Stop loss	Gh_D09G1081	3	A1478T	X493L
Stop gain	Gh_D09G1195	7	G646T	E216X
Stop loss	Gh_D09G1251	4	G348C	X116Y
Stop gain	Gh_D09G1380	1	C44A	S15X
Stop gain	Gh_D09G1482	2	G205T	E69X
Stop gain	Gh_D09G1600	1	C1256A	S419X
Stop loss	Gh_D09G1638	1	T1258G	X420G
Stop gain	Gh_D09G1718	1	C159A	Y53X
Stop gain	Gh_D09G2174	2	C1366T	R456X
Stop gain	Gh_D09G2192	2	A688T	K230X
Stop loss	Gh_D09G2268	6	T1261A	X421R
Stop gain	Gh_D09G2380	12	G1466A	W489X
Stop gain	Gh_D10G0118	3	C400T	Q134X
Stop gain	Gh_D10G0147	4	C329G	S110X

Stop gain	Gh_D10G0267	8	G841T	E281X
Stop gain	Gh_D10G0413	3	C958T	R320X
Stop gain	Gh_D10G0463	22	C2307A	Y769X
Stop gain	Gh_D10G0467	1	G86A	W29X
Stop gain	Gh_D10G0594	1	C758A	S253X
Stop loss	Gh_D10G0600	5	T1618C	X540Q
Stop gain	Gh_D10G0635	1	C67T	Q23X
Stop gain	Gh_D10G0725	17	C2014T	Q672X
Stop loss	Gh_D10G1097	2	T616C	X206Q
Stop gain	Gh_D10G1098	1	C55T	Q19X
Stop gain	Gh_D10G1272	1	G329A	W110X
Stop gain	Gh_D10G1281	2	C289T	Q97X
Stop loss	Gh_D10G1432	13	T1666C	X556Q
Stop gain	Gh_D10G1580	2	C115T	Q39X
Stop gain	Gh_D10G1940	2	C3244T	Q1082X
Stop gain	Gh_D10G1944	3	C1821G	Y607X
Stop gain	Gh_D10G1944	1	C325T	Q109X
Stop loss	Gh_D10G1949	5	T1171C	X391R
Stop loss	Gh_D10G1962	1	A159C	X53C
Stop gain	Gh_D10G2175	1	C214T	Q72X
Stop gain	Gh_D10G2208	1	G95A	W32X
Stop gain	Gh_D10G2208	1	G163T	E55X
Stop loss	Gh_D10G2209	2	T1300C	X434R
Stop gain	Gh_D10G2263	8	C5560T	Q1854X
Stop loss	Gh_D10G2271	1	T1120G	X374E
Stop gain	Gh_D10G2274	3	C436T	Q146X
Stop gain	Gh_D10G2278	1	A598T	K200X
Stop gain	Gh_D11G0101	3	C836A	S279X
Stop gain	Gh_D11G0131	4	C402A	Y134X
Stop gain	Gh_D11G0225	2	C162G	Y54X
Stop loss	Gh_D11G0270	2	T826G	X276E
Stop loss	Gh_D11G0270	2	A827G	X276W
Stop gain	Gh_D11G0293	1	G340T	G114X
Stop gain	Gh_D11G0489	5	G436T	E146X
Stop gain	Gh_D11G0528	9	G1201T	E401X
Stop gain	Gh_D11G0653	1	A904T	K302X
Stop gain	Gh_D11G0745	3	C187T	Q63X
Stop gain	Gh_D11G0799	4	G2465A	W822X
Stop gain	Gh_D11G0799	1	C834A	C278X
Stop gain	Gh_D11G0851	4	G340T	E114X
Stop gain	Gh_D11G0960	6	G1405T	E469X
Stop gain	Gh_D11G1066	1	T186A	Y62X
Stop gain	Gh_D11G1080	1	G52T	E18X
Stop gain	Gh_D11G1084	12	G1660T	E554X
Stop gain	Gh_D11G1084	13	C2260T	Q754X
Stop gain	Gh_D11G1086	19	T2946A	C982X
Stop loss	Gh_D11G1101	1	A864G	X288W
Stop gain	Gh_D11G1159	2	T257A	L86X

Stop gain	Gh_D11G1180	1	G279A	W93X
Stop gain	Gh_D11G1196	8	A1630T	K544X
Stop gain	Gh_D11G1202	1	C81A	Y27X
Stop gain	Gh_D11G1418	1	C118T	Q40X
Stop loss	Gh_D11G1428	6	T2032C	X678Q
Stop gain	Gh_D11G1431	6	G1886A	W629X
Stop gain	Gh_D11G1433	1	G238T	E80X
Stop loss	Gh_D11G1650	1	T1705C	X569Q
Stop loss	Gh_D11G1651	4	G1485T	X495Y
Stop loss	Gh_D11G1952	1	T760A	X254K
Stop loss	Gh_D11G2144	1	T781C	X261Q
Stop loss	Gh_D11G2145	1	T1246C	X416Q
Stop gain	Gh_D11G2146	3	C1222T	Q408X
Stop gain	Gh_D11G2240	3	G343T	G115X
Stop gain	Gh_D11G2335	1	C700T	Q234X
Stop gain	Gh_D11G2344	1	C673T	R225X
Stop gain	Gh_D11G2639	2	A211T	K71X
Stop gain	Gh_D11G2642	1	C498A	Y166X
Stop gain	Gh_D11G2678	2	G378A	W126X
Stop gain	Gh_D11G2891	3	C688T	Q230X
Stop gain	Gh_D11G2910	4	A1009T	K337X
Stop gain	Gh_D11G2924	3	T1701G	Y567X
Stop loss	Gh_D11G3016	1	A875C	X292S
Stop gain	Gh_D11G3113	11	C6496T	R2166X
Stop gain	Gh_D11G3113	8	G5035T	E1679X
Stop loss	Gh_D11G3133	1	G239C	X80S
Stop gain	Gh_D11G3160	1	C293A	S98X
Stop gain	Gh_D11G3324	1	G281A	W94X
Stop gain	Gh_D12G0109	2	G883T	E295X
Stop gain	Gh_D12G0210	2	A934T	K312X
Stop gain	Gh_D12G0211	1	G437A	W146X
Stop gain	Gh_D12G0425	1	G328T	E110X
Stop gain	Gh_D12G0526	1	C124T	Q42X
Stop loss	Gh_D12G1272	2	G197C	X66S
Stop gain	Gh_D12G1444	4	C1399T	Q467X
Stop gain	Gh_D12G1493	3	G293A	W98X
Stop gain	Gh_D12G1523	7	C931T	R311X
Stop gain	Gh_D12G1937	1	C79T	Q27X
Stop gain	Gh_D12G2020	11	T1017G	Y339X
Stop gain	Gh_D12G2062	2	T480A	Y160X
Stop loss	Gh_D12G2131	1	T772A	X258R
Stop gain	Gh_D12G2471	1	C436T	R146X
Stop gain	Gh_D12G2485	3	G687A	W229X
Stop gain	Gh_D12G2524	2	G1295A	W432X
Stop gain	Gh_D12G2528	5	C767A	S256X
Stop gain	Gh_D12G2571	6	G874T	G292X
Stop gain	Gh_D13G0064	2	C1066T	Q356X
Stop loss	Gh_D13G0065	4	T826C	X276Q

Stop gain	Gh_D13G0066	1	T1580A	L527X
Stop gain	Gh_D13G0082	1	G834A	W278X
Stop gain	Gh_D13G0089	14	C2314T	Q772X
Stop loss	Gh_D13G0106	2	T262A	X88K
Stop gain	Gh_D13G0140	1	T329A	L110X
Stop gain	Gh_D13G0527	1	G31T	E11X
Stop gain	Gh_D13G0569	3	C652T	Q218X
Stop gain	Gh_D13G0612	2	C327G	Y109X
Stop gain	Gh_D13G0613	1	C239A	S80X
Stop gain	Gh_D13G0690	1	C883T	Q295X
Stop loss	Gh_D13G0690	1	A1149G	X383W
Stop loss	Gh_D13G0708	2	G836T	X279L
Stop gain	Gh_D13G0750	5	C823T	Q275X
Stop gain	Gh_D13G0872	3	C178T	R60X
Stop gain	Gh_D13G1058	2	G478T	E160X
Stop loss	Gh_D13G1248	5	G810T	X270Y
Stop gain	Gh_D13G1350	7	C1386A	C462X
Stop gain	Gh_D13G1611	2	G374A	W125X
Stop gain	Gh_D13G1616	2	T455A	L152X
Stop gain	Gh_D13G1710	1	G51A	W17X
Stop gain	Gh_D13G1739	10	G919T	E307X
Stop gain	Gh_D13G1743	1	T413A	L138X
Stop gain	Gh_D13G1799	1	C29A	S10X
Stop loss	Gh_D13G1870	1	T277C	X93Q
Stop loss	Gh_D13G1926	3	T1201C	X401Q
Stop gain	Gh_D13G1980	11	G1274A	W425X
Stop gain	Gh_D13G2047	1	G43T	G15X
Stop gain	Gh_D13G2125	2	C1124G	S375X
Stop loss	Gh_D13G2148	5	A1407G	X469W
Stop gain	Gh_D13G2161	1	G304T	G102X
Stop gain	Gh_D13G2163	3	G701A	W234X
Stop gain	Gh_D13G2184	1	C304T	Q102X
Stop loss	Gh_D13G2295	3	A582G	X194W
Stop loss	Gh_A03G1993	1	T910C	X304Q
Stop gain	Gh_A03G2010	8	C989G	S330X
Stop gain	Gh_A03G2021	1	C988T	Q330X
Stop gain	Gh_A03G2044	1	G110A	W37X
Stop gain	Gh_A03G2071	2	G275A	W92X
Stop gain	Gh_A03G2074	2	C1602A	Y534X
Stop loss	Gh_A03G2088	1	T403C	X135R
Stop loss	Gh_A03G2120	1	T502C	X168R
Stop gain	Gh_A03G2132	5	G728A	W243X
Stop gain	Gh_A04G1455	1	C47A	S16X
Stop gain	Gh_A05G3627	1	C296A	S99X
Stop gain	Gh_A05G3711	1	T626G	L209X
Stop gain	Gh_A07G2186	1	C565T	R189X
Stop loss	Gh_A07G2199	21	G4676C	X1559S
Stop gain	Gh_A07G2232	2	C622T	Q208X

Stop gain	Gh_A07G2298	13	C2201G	S734X
Stop loss	Gh_A07G2317	3	T508C	X170Q
Stop loss	Gh_A07G2320	3	T1006A	X336K
Stop gain	Gh_A07G2326	1	G138A	W46X
Stop gain	Gh_A08G2298	3	A1165T	K389X
Stop gain	Gh_A08G2412	2	C184T	Q62X
Stop gain	Gh_A09G2290	3	G1068A	W356X
Stop gain	Gh_A09G2359	7	C907T	R303X
Stop gain	Gh_A09G2444	1	C145T	Q49X
Stop gain	Gh_A09G2482	3	C377A	S126X
Stop gain	Gh_A09G2530	1	C7T	Q3X
Stop gain	Gh_A11G3084	3	A100T	K34X
Stop gain	Gh_A11G3140	1	C121T	Q41X
Stop loss	Gh_A11G3153	1	T541C	X181Q
Stop gain	Gh_A11G3245	1	C7T	Q3X
Stop loss	Gh_A12G2527	1	A423C	X141C
Stop gain	Gh_A12G2533	2	C152A	S51X
Stop gain	Gh_A12G2565	1	G14A	W5X
Stop loss	Gh_A12G2705	1	A1085T	X362L
Stop gain	Gh_A13G2228	2	G291A	W97X
Stop loss	Gh_A13G2314	8	T1303G	X435E
Stop gain	Gh_A13G2318	1	C1234T	R412X
Stop gain	Gh_A13G2321	1	C355T	Q119X
Stop gain	Gh_A13G2349	5	C791G	S264X
Stop gain	Gh_D01G2349	3	C349T	R117X
Stop gain	Gh_D01G2368	1	C46T	Q16X
Stop gain	Gh_D03G1744	7	C763T	Q255X
Stop gain	Gh_D03G1784	6	G767A	W256X
Stop gain	Gh_D04G1993	1	C94T	R32X
Stop gain	Gh_D04G1995	1	C303G	Y101X
Stop gain	Gh_D07G2373	1	G208T	G70X
Stop gain	Gh_D07G2373	1	C175T	Q59X
Stop gain	Gh_D07G2441	1	C43T	Q15X
Stop gain	Gh_D08G2655	4	T779G	L260X
Stop loss	Gh_D08G2749	1	G992C	X331S
Stop gain	Gh_D09G2437	3	C194A	S65X
Stop gain	Gh_D10G2446	6	C1318T	R440X
Stop gain	Gh_D11G3390	1	T809A	L270X
Stop loss	Gh_D11G3392	19	T2248C	X750R
Stop gain	Gh_D11G3392	16	G1670A	W557X
Stop gain	Gh_D11G3437	1	C199T	Q67X
Stop gain	Gh_D11G3465	3	C334T	Q112X
Stop gain	Gh_D12G2652	2	G410A	W137X
Stop loss	Gh_D12G2799	3	A326C	X109S
Stop gain	Gh_D12G2826	2	C1005G	Y335X
Stop gain	Gh_Sca004717G07	4	G1936T	E646X
Stop gain	Gh_Sca004717G10	1	T450G	Y150X
Stop gain	Gh_Sca004801G01	2	A4069T	K1357X

Stop gain	Gh_Sca004821G02	1	G918A	W306X
Stop gain	Gh_Sca004842G09	2	C127T	R43X
Stop loss	Gh_Sca004965G03	14	T5488A	X1830R
Stop gain	Gh_Sca005059G03	1	C5A	S2X
Stop gain	Gh_Sca005199G01	5	C385T	Q129X
Stop gain	Gh_Sca005232G01	1	T416A	L139X
Stop loss	Gh_Sca005300G03	1	A411G	X137W
Stop gain	Gh_Sca005537G02	2	C703T	R235X
Stop gain	Gh_Sca005755G01	2	C1232G	S411X
Stop loss	Gh_Sca006711G02	1	T358C	X120R
Stop gain	Gh_Sca007090G01	1	G17A	W6X
Stop gain	Gh_Sca007876G01	1	C397T	Q133X
Stop gain	Gh_Sca007900G01	1	G65A	W22X
Stop loss	Gh_Sca008391G01	1	A288C	X96Y
Stop gain	Gh_Sca008554G01	1	C315A	Y105X
Stop gain	Gh_Sca010075G01	1	C180G	Y60X
Stop gain	Gh_Sca018371G01	1	G416A	W139X
Stop gain	Gh_Sca150570G01	1	G185A	W62X
Stop gain	Gh_Sca150570G01	1	T102A	Y34X
