# Supplementary material for: Abundant Small Genetic Alterations after Upland Cotton Domestication
Source: Biomed Res Int. 2018 Dec 18;2018:9254302. doi: 10.1155/2018/9254302 (PMC6312614; doi:10.1155/2018/9254302)
Supplement: Supplementary 2 — Table S2: frame shift and nonsense indel mutations between the cultivated and wild cottons. [file 9254302.f2.pdf]

TABLE S2: Frameshift and terminator changes derived by InDel mutations between the cultivated and wild cottons

Category	Gene	Exon	InDel variation cultivated/wild
Frame shift	Gh_A01G0035	11	1076_1077insACTCGATTCA
Frame shift	Gh_A01G0110	1	265delC
Frame shift	Gh_A01G0111	5	1231_1232insAA
Frame shift	Gh_A01G0136	1	13delT
Frame shift	Gh_A01G0153	4	379_380insA
Frame shift	Gh_A01G0175	1	1_2insA
Frame shift	Gh_A01G0285	4	1803delA
Frame shift	Gh_A01G0286	13	1837_1840del
Frame shift	Gh_A01G0307	2	304_305del
Frame shift	Gh_A01G0315	4	2495delA
Frame shift	Gh_A01G0545	4	992_993insTTGC
Frame shift	Gh_A01G0687	3	1259_1260del
Frame shift	Gh_A01G0695	1	9_10insAT
Frame shift	Gh_A01G0840	4	539_540insA
Frame shift	Gh_A01G0840	5	748_775del
Frame shift	Gh_A01G0846	1	781_782insA
Frame shift	Gh_A01G0915	1	56delT
Frame shift	Gh_A01G0948	6	912_913insT
Frame shift	Gh_A01G1027	8	1105delG
Frame shift	Gh_A01G1027	8	1162_1163insA
Frame shift	Gh_A01G1073	1	853_854insA
Frame shift	Gh_A01G1268	9	1767delT
Frame shift	Gh_A01G1337	4	347_348insC
Frame shift	Gh_A01G1363	1	116delC
Frame shift	Gh_A01G1492	2	89delT
Frame shift	Gh_A01G1625	3	390_391insT
Frame shift	Gh_A01G1741	2	309_310insG
Frame shift	Gh_A01G1849	3	354_357del
Frame shift	Gh_A01G1867	10	1356_1368del
Frame shift	Gh_A01G1872	3	613delA
Frame shift	Gh_A01G1957	1	2566_2567insC
Frame shift	Gh_A01G1982	1	138delC
Frame shift	Gh_A01G1992	12	1461delG
Frame shift	Gh_A01G2026	2	285_286insACCGGAAACAACA
Frame shift	Gh_A01G2107	1	164delA
Frame shift	Gh_A01G2112	1	684delC
Frame shift	Gh_A01G2121	14	1886delA
Frame shift	Gh_A02G0008	1	96_97insTTTACCCGATGCCACC
Frame shift	Gh_A02G0046	1	1293_1294insA
Frame shift	Gh_A02G0103	13	4703delA
Frame shift	Gh_A02G0105	1	709_751del
Frame shift	Gh_A02G0107	1	1217delA
Frame shift	Gh_A02G0107	2	1286delT
Frame shift	Gh_A02G0114	2	1383_1384insGAGAGAGAGA

Frame shift	Gh_A02G0120	2	906delG
Frame shift	Gh_A02G0128	3	1225_1226insT
Frame shift	Gh_A02G0153	1	1_2insAA
Frame shift	Gh_A02G0173	2	228_249del
Frame shift	Gh_A02G0179	5	627_666del
Frame shift	Gh_A02G0186	23	3613_3614insT
Frame shift	Gh_A02G0248	Whole gene	
Frame shift	Gh_A02G0249	2	240delT
Frame shift	Gh_A02G0250	1	180_181del
Frame shift	Gh_A02G0250	1	246delC
Frame shift	Gh_A02G0262	2	311delT
Frame shift	Gh_A02G0421	1	41delT
Frame shift	Gh_A02G0431	1	1129delG
Frame shift	Gh_A02G0431	1	1795delG
Frame shift	Gh_A02G0504	2	335_336insAT
Frame shift	Gh_A02G0607	2	593_594insA
Frame shift	Gh_A02G0615	2	123delT
Frame shift	Gh_A02G0616	4	1249_1250del
Frame shift	Gh_A02G0761	15	1083delT
Frame shift	Gh_A02G0855	1	119_120insGAGG
Frame shift	Gh_A02G0913	5	635delT
Frame shift	Gh_A02G0916	1	487delT
Frame shift	Gh_A02G0917	3	466_500del
Frame shift	Gh_A02G0923	2	358_359del
Frame shift	Gh_A02G0963	4	503_504insGTTGG
Frame shift	Gh_A02G1258	1	105_106insG
Frame shift	Gh_A02G1301	2	292_293del
Frame shift	Gh_A02G1319	1	125_128del
Frame shift	Gh_A02G1321	7	925_926insG
Frame shift	Gh_A02G1402	8	1996delA
Frame shift	Gh_A02G1434	2	247_248insCTATG
Frame shift	Gh_A02G1489	3	1305_1306insT
Frame shift	Gh_A02G1535	1	105_106insT
Frame shift	Gh_A02G1552	1	493_494insG
Frame shift	Gh_A02G1608	18	1268_1269insTT
Frame shift	Gh_A02G1724	4	349delC
Frame shift	Gh_A02G1738	1	6delG
Frame shift	Gh_A03G0048	6	1396delT
Frame shift	Gh_A03G0055	9	915_921del
Frame shift	Gh_A03G0062	1	229_230insA
Frame shift	Gh_A03G0076	2	645delT
Frame shift	Gh_A03G0076	2	740delA
Frame shift	Gh_A03G0090	1	71delC
Frame shift	Gh_A03G0164	3	447_448insG
Frame shift	Gh_A03G0200	4	427delA
Frame shift	Gh_A03G0307	1	48delA
Frame shift	Gh_A03G0339	1	59_60insCC
Frame shift	Gh_A03G0340	1	1_2insA
Frame shift	Gh_A03G0362	3	207_208insAT

Frame shift	Gh_A03G0438	4	642delG
Frame shift	Gh_A03G0450	1	1067_1068insA
Frame shift	Gh_A03G0510	3	1043_1044insA
Frame shift	Gh_A03G0530	5	645_646insT
Frame shift	Gh_A03G0532	5	541_542insT
Frame shift	Gh_A03G0601	7	1719_1722del
Frame shift	Gh_A03G0611	16	2263_2264insA
Frame shift	Gh_A03G0635	1	896_897insT
Frame shift	Gh_A03G0673	2	420_421insTACTTTCTCCGACAAAGCAATATCAATCG
Frame shift	Gh_A03G0688	2	169delG
Frame shift	Gh_A03G0692	2	134delA
Frame shift	Gh_A03G0697	3	1846_1847insC
Frame shift	Gh_A03G0737	8	1637_1638insA
Frame shift	Gh_A03G0765	4	622_623insC
Frame shift	Gh_A03G0792	8	981_982del
Frame shift	Gh_A03G0800	1	263_291del
Frame shift	Gh_A03G0802	1	5delA
Frame shift	Gh_A03G0813	9	1557delT
Frame shift	Gh_A03G0862	10	1214_1215insCTTT
Frame shift	Gh_A03G0875	2	1502_1514del
Frame shift	Gh_A03G0885	2	186delT
Frame shift	Gh_A03G0898	1	28delA
Frame shift	Gh_A03G0988	1	231_232insA
Frame shift	Gh_A03G1051	3	136_137del
Frame shift	Gh_A03G1056	2	109delA
Frame shift	Gh_A03G1073	1	454delC
Frame shift	Gh_A03G1176	1	93delC
Frame shift	Gh_A03G1180	1	406_407insG
Frame shift	Gh_A03G1229	3	277_278insT
Frame shift	Gh_A03G1250	3	535_536insCA
Frame shift	Gh_A03G1259	3	593delG
Frame shift	Gh_A03G1272	3	1763_1764insT
Frame shift	Gh_A03G1278	1	148_149insA
Frame shift	Gh_A03G1298	1	78_79insC
Frame shift	Gh_A03G1327	1	108delT
Frame shift	Gh_A03G1363	1	703delG
Frame shift	Gh_A03G1410	1	293_294del
Frame shift	Gh_A03G1420	2	471delG
Frame shift	Gh_A03G1438	6	1668_1669insTTTTTGTTTCC
Frame shift	Gh_A03G1581	2	228_229insT
Frame shift	Gh_A03G1581	5	445_452del
Frame shift	Gh_A03G1635	1	1352_1353insCG
Frame shift	Gh_A03G1788	1	11_12insA
Frame shift	Gh_A03G1829	1	1096_1099del
Frame shift	Gh_A03G1833	1	67_68insCC
Frame shift	Gh_A03G1839	1	254delT
Frame shift	Gh_A03G1875	1	91delG
Frame shift	Gh_A03G1938	5	329_330insGC

Frame shift	Gh_A03G1962	3	1355_1356insT
Frame shift	Gh_A03G1994	3	1092_1093insG
Frame shift	Gh_A03G1995	2	964_977del
Frame shift	Gh_A03G2010	8	1192_1193del
Frame shift	Gh_A03G2011	1	243_244insT
Frame shift	Gh_A03G2045	2	137_138insA
Frame shift	Gh_A03G2081	14	1464_1465del
Frame shift	Gh_A03G2104	6	1862_1863insAT
Frame shift	Gh_A03G2132	5	630delT
Frame shift	Gh_A04G0056	1	71_72insTT
Frame shift	Gh_A04G0075	1	92delT
Frame shift	Gh_A04G0096	1	145delC
Frame shift	Gh_A04G0133	3	368delT
Frame shift	Gh_A04G0167	1	38_39insA
Frame shift	Gh_A04G0183	4	941_942insAA
Frame shift	Gh_A04G0187	1	1653_1662del
Frame shift	Gh_A04G0189	2	44_45insT
Frame shift	Gh_A04G0217	15	1307_1308insT
Frame shift	Gh_A04G0224	1	220_221insTG
Frame shift	Gh_A04G0229	1	31_56del
Frame shift	Gh_A04G0269	3	1099_1109del
Frame shift	Gh_A04G0272	2	1083_1084insA
Frame shift	Gh_A04G0297	3	1096_1097insT
Frame shift	Gh_A04G0566	9	1119delC
Frame shift	Gh_A04G0589	7	2226_2227insT
Frame shift	Gh_A04G0589	7	2123_2124insAA
Frame shift	Gh_A04G0708	3	258delG
Frame shift	Gh_A04G0849	3	262_263insTT
Frame shift	Gh_A04G0885	1	201_202insA
Frame shift	Gh_A04G0975	5	680_681insC
Frame shift	Gh_A04G1007	1	1delA
Frame shift	Gh_A04G1011	4	298delG
Frame shift	Gh_A04G1081	1	640_643del
Frame shift	Gh_A04G1215	1	56_93del
Frame shift	Gh_A04G1283	1	165delG
Frame shift	Gh_A04G1290	2	445delT
Frame shift	Gh_A05G0017	11	897_898insG
Frame shift	Gh_A05G0080	4	750_751insGGCCAAAAGGT
Frame shift	Gh_A05G0080	5	827_828del
Frame shift	Gh_A05G0188	1	83_84insC
Frame shift	Gh_A05G0230	2	1309_1310insCA
Frame shift	Gh_A05G0230	2	1356_1357insT
Frame shift	Gh_A05G0230	2	1521_1524del
Frame shift	Gh_A05G0408	2	344delA
Frame shift	Gh_A05G0474	3	123_124insTCTAACT
Frame shift	Gh_A05G0514	9	1023delA
Frame shift	Gh_A05G0527	3	4975_4997del
Frame shift	Gh_A05G0661	3	459delT

Frame shift	Gh_A05G0666	8	914_915insT
Frame shift	Gh_A05G0682	1	436_437insTA
Frame shift	Gh_A05G0714	1	406delA
Frame shift	Gh_A05G0776	1	118_119insC
Frame shift	Gh_A05G0927	2	120delC
Frame shift	Gh_A05G1096	2	524_525insC
Frame shift	Gh_A05G1097	1	82_83insC
Frame shift	Gh_A05G1118	12	1247_1250del
Frame shift	Gh_A05G1118	17	1927_1928insA
Frame shift	Gh_A05G1306	3	431_432insAT
Frame shift	Gh_A05G1314	3	623delA
Frame shift	Gh_A05G1324	1	385delC
Frame shift	Gh_A05G1379	2	609_610insA
Frame shift	Gh_A05G1400	2	333_334insG
Frame shift	Gh_A05G1403	11	1266_1266del
Frame shift	Gh_A05G1560	1	519delA
Frame shift	Gh_A05G1681	1	347_348insG
Frame shift	Gh_A05G1769	1	461_462insA
Frame shift	Gh_A05G1899	3	322_323insG
Frame shift	Gh_A05G1943	3	597_598del
Frame shift	Gh_A05G2018	19	5378_5379insC
Frame shift	Gh_A05G2037	2	371delA
Frame shift	Gh_A05G2061	1	174_175insC
Frame shift	Gh_A05G2097	1	23_24insT
Frame shift	Gh_A05G2102	4	1058delC
Frame shift	Gh_A05G2149	3	66_67insC
Frame shift	Gh_A05G2210	1	219_220insG
Frame shift	Gh_A05G2493	6	2878_2879insA
Frame shift	Gh_A05G2558	2	117_118insATATTTTTTCAGG
Frame shift	Gh_A05G2582	2	511_512insA
Frame shift	Gh_A05G2584	3	635_641del
Frame shift	Gh_A05G2776	4	807_808insGT
Frame shift	Gh_A05G2850	1	226_227del
Frame shift	Gh_A05G2850	3	904_905insG
Frame shift	Gh_A05G2896	7	1125delT
Frame shift	Gh_A05G2903	3	306delT
Frame shift	Gh_A05G3113	5	823_824del
Frame shift	Gh_A05G3154	2	542delG
Frame shift	Gh_A05G3211	2	783_784insTTCTA
Frame shift	Gh_A05G3263	2	424delT
Frame shift	Gh_A05G3343	1	769delC
Frame shift	Gh_A05G3343	1	798_801del
Frame shift	Gh_A05G3343	1	2174_2175insA
Frame shift	Gh_A05G3504	5	459delG
Frame shift	Gh_A05G3562	4	453delA
Frame shift	Gh_A05G3578	2	11delA
Frame shift	Gh_A05G3623	1	212_227del
Frame shift	Gh_A05G3885	2	232_236del

Frame shift	Gh_A05G3969	1	1013_1014insTGAGATG
Frame shift	Gh_A06G0006	2	26_27insG
Frame shift	Gh_A06G0117	1	940delC
Frame shift	Gh_A06G0119	2	1383delC
Frame shift	Gh_A06G0138	6	925delA
Frame shift	Gh_A06G0138	6	684_685del
Frame shift	Gh_A06G0148	6	762_763insA
Frame shift	Gh_A06G0152	1	234_235insAAAAACCTTGT
Frame shift	Gh_A06G0174	2	154_155insT
Frame shift	Gh_A06G0194	2	380delC
Frame shift	Gh_A06G0207	1	274delG
Frame shift	Gh_A06G0272	11	1083delT
Frame shift	Gh_A06G0275	1	930delT
Frame shift	Gh_A06G0275	1	1043_1058del
Frame shift	Gh_A06G0292	3	2432delT
Frame shift	Gh_A06G0293	1	709delG
Frame shift	Gh_A06G0348	3	610_611insA
Frame shift	Gh_A06G0433	9	1582_1583insAG
Frame shift	Gh_A06G0438	1	263delA
Frame shift	Gh_A06G0505	4	881delG
Frame shift	Gh_A06G0539	3	1366delT
Frame shift	Gh_A06G0623	15	1328_1329insA
Frame shift	Gh_A06G0638	14	1821_1822del
Frame shift	Gh_A06G0639	1	466delC
Frame shift	Gh_A06G0646	3	431_432insGGTTTGT
Frame shift	Gh_A06G0671	4	792_793del
Frame shift	Gh_A06G0676	1	1_2insAA
Frame shift	Gh_A06G0690	4	396_397insG
Frame shift	Gh_A06G0704	5	825_829del
Frame shift	Gh_A06G0708	3	504_505insA
Frame shift	Gh_A06G0728	1	494_495del
Frame shift	Gh_A06G0845	3	535_536insC
Frame shift	Gh_A06G0874	9	1803_1803del
Frame shift	Gh_A06G0897	2	273_274insT
Frame shift	Gh_A06G0922	6	1162_1163del
Frame shift	Gh_A06G0991	24	2907_2908insG
Frame shift	Gh_A06G1001	8	1021_1022insC
Frame shift	Gh_A06G1065	3	701_702del
Frame shift	Gh_A06G1066	3	831delA
Frame shift	Gh_A06G1098	1	613_628del
Frame shift	Gh_A06G1118	Whole gene	
Frame shift	Gh_A06G1121	7	2747_2748insC
Frame shift	Gh_A06G1134	1	321_325del
Frame shift	Gh_A06G1142	1	1455_1456insG
Frame shift	Gh_A06G1192	3	354_355insA
Frame shift	Gh_A06G1210	3	98_99insT
Frame shift	Gh_A06G1337	1	652_653insT
Frame shift	Gh_A06G1494	3	139_140insG

Frame shift	Gh_A06G1526	1	136delA
Frame shift	Gh_A06G1533	2	1497delG
Frame shift	Gh_A06G1552	18	1252_1253del
Frame shift	Gh_A06G1558	1	1356delA
Frame shift	Gh_A06G1592	1	214delG
Frame shift	Gh_A06G1595	8	735delC
Frame shift	Gh_A06G1619	1	21delA
Frame shift	Gh_A06G1637	2	178delT
Frame shift	Gh_A06G1649	4	1369delA
Frame shift	Gh_A06G1651	1	665_666insTCAAGAT
Frame shift	Gh_A06G1670	1	238delA
Frame shift	Gh_A06G1681	2	239_240insG
Frame shift	Gh_A06G1691	2	276_277insGCACCATTCAACGATC
Frame shift	Gh_A06G1692	4	523delA
Frame shift	Gh_A06G1693	12	1038_1039insGAGT
Frame shift	Gh_A06G1701	3	734delA
Frame shift	Gh_A06G1753	2	195delA
Frame shift	Gh_A06G1762	1	80_81del
Frame shift	Gh_A06G1803	5	1579_1580del
Frame shift	Gh_A06G1915	7	513_516del
Frame shift	Gh_A06G1919	1	680_681insA
Frame shift	Gh_A06G1919	1	635delC
Frame shift	Gh_A06G1951	Whole gene	
Frame shift	Gh_A06G2001	1	889_895del
Frame shift	Gh_A06G2029	2	26delA
Frame shift	Gh_A06G2036	6	1125delT
Frame shift	Gh_A06G2043	1	63_64insT
Frame shift	Gh_A06G2055	1	76_77insGGTGTGGCTTTCGAGA
Frame shift	Gh_A06G2055	1	1462_1463insG
Frame shift	Gh_A07G0001	2	108delA
Frame shift	Gh_A07G0030	1	650_651insG
Frame shift	Gh_A07G0111	4	1281delA
Frame shift	Gh_A07G0192	1	73_89del
Frame shift	Gh_A07G0193	6	1576delG
Frame shift	Gh_A07G0201	1	433_434insG
Frame shift	Gh_A07G0211	2	224_225insTTGGAAA
Frame shift	Gh_A07G0359	12	2259delA
Frame shift	Gh_A07G0411	1	159_160insG
Frame shift	Gh_A07G0433	1	342_343insT
Frame shift	Gh_A07G0451	1	825_826insC
Frame shift	Gh_A07G0479	1	293delC
Frame shift	Gh_A07G0518	1	1605_1606insA
Frame shift	Gh_A07G0527	1	901_919del
Frame shift	Gh_A07G0528	2	361delG
Frame shift	Gh_A07G0575	6	777_784del
Frame shift	Gh_A07G0593	7	552_553del
Frame shift	Gh_A07G0600	2	76_77insC
Frame shift	Gh_A07G0602	1	452delT

Frame shift	Gh_A07G0632	3	894delG
Frame shift	Gh_A07G0632	5	1636_1637insA
Frame shift	Gh_A07G0657	1	40delG
Frame shift	Gh_A07G0666	1	110_111insA
Frame shift	Gh_A07G0693	2	1083_1084insC
Frame shift	Gh_A07G0720	5	419_420insC
Frame shift	Gh_A07G0823	2	70_73del
Frame shift	Gh_A07G0837	2	373_374insT
Frame shift	Gh_A07G0839	Whole gene	
Frame shift	Gh_A07G1006	5	989_990del
Frame shift	Gh_A07G1055	5	1115_1116insC
Frame shift	Gh_A07G1278	1	87_88del
Frame shift	Gh_A07G1292	4	306_307insA
Frame shift	Gh_A07G1306	2	122delG
Frame shift	Gh_A07G1322	3	900_901del
Frame shift	Gh_A07G1341	1	351_352insG
Frame shift	Gh_A07G1373	1	13_14insA
Frame shift	Gh_A07G1396	1	641delG
Frame shift	Gh_A07G1400	2	1285_1286del
Frame shift	Gh_A07G1401	1	226delA
Frame shift	Gh_A07G1405	1	289_308del
Frame shift	Gh_A07G1410	1	140_141insT
Frame shift	Gh_A07G1437	2	174_175insG
Frame shift	Gh_A07G1503	4	898_899insA
Frame shift	Gh_A07G1506	1	633_634del
Frame shift	Gh_A07G1526	2	1028delT
Frame shift	Gh_A07G1539	1	121delT
Frame shift	Gh_A07G1552	Whole gene	
Frame shift	Gh_A07G1558	1	17_18insTTTCCATAAG
Frame shift	Gh_A07G1572	1	1200_1201del
Frame shift	Gh_A07G1619	12	1663_1664insGT
Frame shift	Gh_A07G1622	1	143_146del
Frame shift	Gh_A07G1630	1	402delG
Frame shift	Gh_A07G1647	1	1431_1437del
Frame shift	Gh_A07G1662	1	1048_1051del
Frame shift	Gh_A07G1733	2	523_524insG
Frame shift	Gh_A07G1778	1	20_21insG
Frame shift	Gh_A07G1783	9	1974delA
Frame shift	Gh_A07G1806	4	179_183del
Frame shift	Gh_A07G1808	1	1_2insA
Frame shift	Gh_A07G1959	7	1413delT
Frame shift	Gh_A07G2017	2	1009_1010insG
Frame shift	Gh_A07G2139	2	441delA
Frame shift	Gh_A07G2163	1	266delA
Frame shift	Gh_A07G2194	3	254_255insT
Frame shift	Gh_A07G2238	3	1208_1209insTGAG
Frame shift	Gh_A07G2285	5	1050_1051insAACTTTC
Frame shift	Gh_A07G2293	1	1_2insA

Frame shift	Gh_A07G2308	1	10_11del
Frame shift	Gh_A07G2332	1	11_12del
Frame shift	Gh_A07G2345	7	618delT
Frame shift	Gh_A08G0028	1	233_234del
Frame shift	Gh_A08G0032	3	2259_2260insTTAGTTGTGCAACAACAACA
Frame shift	Gh_A08G0042	4	619_620insA
Frame shift	Gh_A08G0044	10	1475_1476insA
Frame shift	Gh_A08G0047	1	762delC
Frame shift	Gh_A08G0068	1	151_152insT
Frame shift	Gh_A08G0096	1	1347_1348insT
Frame shift	Gh_A08G0099	2	486_487insG
Frame shift	Gh_A08G0119	1	59delC
Frame shift	Gh_A08G0140	2	96delT
Frame shift	Gh_A08G0185	3	2576delA
Frame shift	Gh_A08G0185	4	3551_3552insC
Frame shift	Gh_A08G0233	1	521_522insAT
Frame shift	Gh_A08G0393	2	1673_1674insC
Frame shift	Gh_A08G0436	2	43delG
Frame shift	Gh_A08G0492	1	8_9insGG
Frame shift	Gh_A08G0553	1	43delC
Frame shift	Gh_A08G0644	3	326delT
Frame shift	Gh_A08G0650	1	5_6insAT
Frame shift	Gh_A08G0865	1	209delA
Frame shift	Gh_A08G0949	1	219delC
Frame shift	Gh_A08G1538	3	342_343insG
Frame shift	Gh_A08G1637	1	25_28del
Frame shift	Gh_A08G1640	9	1318_1319insTA
Frame shift	Gh_A08G1649	2	973_991del
Frame shift	Gh_A08G1745	2	254_255insG
Frame shift	Gh_A08G1810	1	482delG
Frame shift	Gh_A08G1824	1	52_53insTCCTTAA
Frame shift	Gh_A08G1881	1	801delT
Frame shift	Gh_A08G1885	1	28delC
Frame shift	Gh_A08G1886	1	1000_1003del
Frame shift	Gh_A08G1899	1	42_45del
Frame shift	Gh_A08G2009	3	429delC
Frame shift	Gh_A08G2048	1	14_15del
Frame shift	Gh_A08G2128	4	407_408insG
Frame shift	Gh_A08G2143	2	252_259del
Frame shift	Gh_A08G2252	5	1721_1722del
Frame shift	Gh_A08G2331	1	238_241del
Frame shift	Gh_A09G0108	1	294_295insC
Frame shift	Gh_A09G0177	1	571_572insG
Frame shift	Gh_A09G0179	2	1144_1145insT
Frame shift	Gh_A09G0186	2	1856delC
Frame shift	Gh_A09G0186	3	2134_2135del
Frame shift	Gh_A09G0191	1	1delA
Frame shift	Gh_A09G0225	4	1043delT

Frame shift	Gh_A09G0233	6	2118_2119insT
Frame shift	Gh_A09G0261	4	2062_2063insC
Frame shift	Gh_A09G0269	3	930_930del
Frame shift	Gh_A09G0270	3	189_190del
Frame shift	Gh_A09G0354	1	79_80insGT
Frame shift	Gh_A09G0511	2	431_432insT
Frame shift	Gh_A09G0535	2	392delC
Frame shift	Gh_A09G0536	19	1794_1795insT
Frame shift	Gh_A09G0742	1	236delA
Frame shift	Gh_A09G0745	1	371delG
Frame shift	Gh_A09G0821	1	2_3insAGTGGCG
Frame shift	Gh_A09G0834	1	40_41insG
Frame shift	Gh_A09G0834	2	195delC
Frame shift	Gh_A09G0834	2	298_299insT
Frame shift	Gh_A09G1043	2	30_55del
Frame shift	Gh_A09G1052	2	684delT
Frame shift	Gh_A09G1085	4	1678_1679insAA
Frame shift	Gh_A09G1148	4	1157_1158insTTTTT
Frame shift	Gh_A09G1186	6	1305_1306insAT
Frame shift	Gh_A09G1190	2	616_617insA
Frame shift	Gh_A09G1205	7	1148_1149insAA
Frame shift	Gh_A09G1205	7	1196_1200del
Frame shift	Gh_A09G1250	6	804delC
Frame shift	Gh_A09G1333	1	79_82del
Frame shift	Gh_A09G1359	1	1374delC
Frame shift	Gh_A09G1372	9	2580_2581insA
Frame shift	Gh_A09G1380	7	636_637insGA
Frame shift	Gh_A09G1408	2	774_777del
Frame shift	Gh_A09G1457	3	1345delA
Frame shift	Gh_A09G1522	20	2953delT
Frame shift	Gh_A09G1537	11	1314_1315insCCCC
Frame shift	Gh_A09G1545	2	1623delG
Frame shift	Gh_A09G1549	2	126delA
Frame shift	Gh_A09G1602	4	668_669insGAAAT
Frame shift	Gh_A09G1634	1	430delT
Frame shift	Gh_A09G1634	1	397delT
Frame shift	Gh_A09G1741	1	177delC
Frame shift	Gh_A09G1846	3	744delA
Frame shift	Gh_A09G1897	2	1070_1071insT
Frame shift	Gh_A09G2021	1	162_163insT
Frame shift	Gh_A09G2091	2	186_187insT
Frame shift	Gh_A09G2208	2	502_503insCC
Frame shift	Gh_A09G2287	2	202delA
Frame shift	Gh_A09G2355	4	1711_1714del
Frame shift	Gh_A09G2422	2	427_433del
Frame shift	Gh_A09G2463	1	231delA
Frame shift	Gh_A09G2525	2	238delA
Frame shift	Gh_A10G0072	2	417_418insC

Frame shift	Gh_A10G0102	1	422_423insT
Frame shift	Gh_A10G0129	4	639_654del
Frame shift	Gh_A10G0134	1	14_15insAG
Frame shift	Gh_A10G0434	3	533delC
Frame shift	Gh_A10G0434	5	954delA
Frame shift	Gh_A10G0501	1	523delC
Frame shift	Gh_A10G0538	1	153_154del
Frame shift	Gh_A10G0554	1	446delA
Frame shift	Gh_A10G0731	5	579delT
Frame shift	Gh_A10G0917	1	150delA
Frame shift	Gh_A10G0938	12	2447delA
Frame shift	Gh_A10G0938	3	476delC
Frame shift	Gh_A10G0941	1	34_35insT
Frame shift	Gh_A10G1005	1	310_311del
Frame shift	Gh_A10G1012	1	1delA
Frame shift	Gh_A10G1129	2	724_725insTA
Frame shift	Gh_A10G1205	7	793_794del
Frame shift	Gh_A10G1481	1	173_174insC
Frame shift	Gh_A10G1541	Whole gene	
Frame shift	Gh_A10G1560	1	657delC
Frame shift	Gh_A10G1616	3	321delA
Frame shift	Gh_A10G1669	1	97delT
Frame shift	Gh_A10G1722	2	1474delA
Frame shift	Gh_A10G1743	1	179delC
Frame shift	Gh_A10G1827	3	120_142del
Frame shift	Gh_A10G1850	14	1883_1914del
Frame shift	Gh_A10G1875	1	1747_1748insC
Frame shift	Gh_A10G1974	5	488_489insC
Frame shift	Gh_A10G2007	6	678_679insA
Frame shift	Gh_A10G2012	1	2_3insAT
Frame shift	Gh_A10G2042	1	418_419insT
Frame shift	Gh_A10G2087	2	563_564insA
Frame shift	Gh_A10G2109	Whole gene	
Frame shift	Gh_A10G2112	1	853_854insA
Frame shift	Gh_A10G2113	2	632_633del
Frame shift	Gh_A10G2286	2	241_242insGGGG
Frame shift	Gh_A10G2357	2	1136delC
Frame shift	Gh_A11G0007	1	11_12insAGAAG
Frame shift	Gh_A11G0189	18	3579_3580insTT
Frame shift	Gh_A11G0281	2	789_790insT
Frame shift	Gh_A11G0284	8	825_826insC
Frame shift	Gh_A11G0296	5	401delT
Frame shift	Gh_A11G0312	2	299delC
Frame shift	Gh_A11G0313	3	910delC
Frame shift	Gh_A11G0343	3	364_365insAT
Frame shift	Gh_A11G0357	4	1648_1649insCC
Frame shift	Gh_A11G0379	1	1_2insA
Frame shift	Gh_A11G0414	1	424_425insT

Frame shift	Gh_A11G0432	4	582delA
Frame shift	Gh_A11G0632	3	215_216insGGGAA
Frame shift	Gh_A11G0644	1	9_13del
Frame shift	Gh_A11G0663	6	756delG
Frame shift	Gh_A11G0679	1	333delC
Frame shift	Gh_A11G0679	1	455_456insT
Frame shift	Gh_A11G0694	1	37delG
Frame shift	Gh_A11G0703	2	572_573insT
Frame shift	Gh_A11G0763	1	111_114del
Frame shift	Gh_A11G0766	8	700_701del
Frame shift	Gh_A11G0849	1	29_42del
Frame shift	Gh_A11G0877	1	837delG
Frame shift	Gh_A11G0899	1	1183delC
Frame shift	Gh_A11G0990	1	349_382del
Frame shift	Gh_A11G0998	1	754delT
Frame shift	Gh_A11G1363	1	131_132insA
Frame shift	Gh_A11G1363	2	650_651insTA
Frame shift	Gh_A11G1406	1	201delA
Frame shift	Gh_A11G1414	1	97_98insAT
Frame shift	Gh_A11G1460	1	4_5del
Frame shift	Gh_A11G1476	1	304_305insC
Frame shift	Gh_A11G1505	1	1delA
Frame shift	Gh_A11G1512	2	260delC
Frame shift	Gh_A11G1515	1	170_171insTT
Frame shift	Gh_A11G1517	1	161delA
Frame shift	Gh_A11G1521	3	218_219del
Frame shift	Gh_A11G1524	3	1355_1356insT
Frame shift	Gh_A11G1526	5	985delT
Frame shift	Gh_A11G1534	1	54_57del
Frame shift	Gh_A11G1568	6	1911delT
Frame shift	Gh_A11G1641	6	653delT
Frame shift	Gh_A11G1641	6	702_703insAA
Frame shift	Gh_A11G1884	2	58delC
Frame shift	Gh_A11G1904	1	766_767del
Frame shift	Gh_A11G1954	3	437_438insC
Frame shift	Gh_A11G1958	4	1819_1820del
Frame shift	Gh_A11G2059	1	82delG
Frame shift	Gh_A11G2101	2	216_217insA
Frame shift	Gh_A11G2152	1	92_93del
Frame shift	Gh_A11G2161	2	860delA
Frame shift	Gh_A11G2184	1	453delC
Frame shift	Gh_A11G2299	3	282_283insG
Frame shift	Gh_A11G2299	Whole gene	
Frame shift	Gh_A11G2559	3	1598_1599del
Frame shift	Gh_A11G2622	3	629delC
Frame shift	Gh_A11G2636	3	783_784insTTTCT
Frame shift	Gh_A11G2636	4	853_854insCACTA
Frame shift	Gh_A11G2680	3	997_998insTT

Frame shift	Gh_A11G2685	2	54delA
Frame shift	Gh_A11G2748	5	1215_1215del
Frame shift	Gh_A11G2779	2	143_144insG
Frame shift	Gh_A11G2783	1	826_827insA
Frame shift	Gh_A11G2904	1	894_895insA
Frame shift	Gh_A11G2925	3	225delG
Frame shift	Gh_A11G2940	2	496delC
Frame shift	Gh_A11G3022	5	875_876del
Frame shift	Gh_A11G3067	6	1673delA
Frame shift	Gh_A11G3076	1	49delG
Frame shift	Gh_A12G0084	Whole gene	
Frame shift	Gh_A12G0250	14	1850_1851insC
Frame shift	Gh_A12G0386	Whole gene	
Frame shift	Gh_A12G0408	4	1556delC
Frame shift	Gh_A12G0447	8	1013delC
Frame shift	Gh_A12G0528	1	170delC
Frame shift	Gh_A12G0534	4	496_497insA
Frame shift	Gh_A12G0536	2	617_621del
Frame shift	Gh_A12G0569	1	219_220insA
Frame shift	Gh_A12G0576	2	343_344insAA
Frame shift	Gh_A12G0627	8	2092delA
Frame shift	Gh_A12G0659	4	268delA
Frame shift	Gh_A12G0729	2	414delC
Frame shift	Gh_A12G0731	3	108_111del
Frame shift	Gh_A12G0740	1	283_284insG
Frame shift	Gh_A12G0777	11	1029_1030insA
Frame shift	Gh_A12G0780	3	364_376del
Frame shift	Gh_A12G0790	6	815delG
Frame shift	Gh_A12G0791	7	1841_1844del
Frame shift	Gh_A12G0812	5	1191delT
Frame shift	Gh_A12G0839	1	955delG
Frame shift	Gh_A12G0842	5	472delA
Frame shift	Gh_A12G0879	8	528delA
Frame shift	Gh_A12G0911	4	756_759del
Frame shift	Gh_A12G0978	1	5_6insG
Frame shift	Gh_A12G0996	5	741_742insT
Frame shift	Gh_A12G1014	1	25_26insT
Frame shift	Gh_A12G1023	5	2938_2941del
Frame shift	Gh_A12G1055	1	973delG
Frame shift	Gh_A12G1117	3	1483_1484insAT
Frame shift	Gh_A12G1120	1	93_94insA
Frame shift	Gh_A12G1134	1	329_330insT
Frame shift	Gh_A12G1147	1	222_223insG
Frame shift	Gh_A12G1189	5	279_280insC
Frame shift	Gh_A12G1281	2	133delC
Frame shift	Gh_A12G1283	6	877_878insA
Frame shift	Gh_A12G1372	6	513delT
Frame shift	Gh_A12G1473	14	1822_1823insT

Frame shift	Gh_A12G1473	18	2885_2886insA
Frame shift	Gh_A12G1504	3	871delC
Frame shift	Gh_A12G1540	1	100_115del
Frame shift	Gh_A12G1591	2	764delT
Frame shift	Gh_A12G1635	1	115_116insT
Frame shift	Gh_A12G1682	2	1455_1456insA
Frame shift	Gh_A12G1726	5	519delA
Frame shift	Gh_A12G1850	1	95delT
Frame shift	Gh_A12G1883	1	156_157insA
Frame shift	Gh_A12G1980	1	47delT
Frame shift	Gh_A12G2017	1	86_108del
Frame shift	Gh_A12G2059	4	830delC
Frame shift	Gh_A12G2368	4	1586_1587insT
Frame shift	Gh_A12G2377	1	270_271insAT
Frame shift	Gh_A12G2434	1	1_2insA
Frame shift	Gh_A12G2538	6	388_389insAAAGAGAAGCTGCTCGAATTGC
Frame shift	Gh_A12G2556	1	1_2insA
Frame shift	Gh_A12G2561	3	864delC
Frame shift	Gh_A12G2569	4	766delC
Frame shift	Gh_A12G2586	4	716_717insG
Frame shift	Gh_A12G2673	1	1delA
Frame shift	Gh_A13G0019	1	5delT
Frame shift	Gh_A13G0073	14	2318_2319insA
Frame shift	Gh_A13G0091	1	908_921del
Frame shift	Gh_A13G0141	1	795delT
Frame shift	Gh_A13G0152	1	39_40insC
Frame shift	Gh_A13G0152	3	506_507insT
Frame shift	Gh_A13G0155	2	194_195insA
Frame shift	Gh_A13G0155	2	323delA
Frame shift	Gh_A13G0173	1	1056_1063del
Frame shift	Gh_A13G0175	1	318_319insA
Frame shift	Gh_A13G0206	1	373delA
Frame shift	Gh_A13G0206	1	390_394del
Frame shift	Gh_A13G0324	3	119_120insC
Frame shift	Gh_A13G0392	1	1_2insA
Frame shift	Gh_A13G0779	3	1989delA
Frame shift	Gh_A13G0802	6	1279_1280insA
Frame shift	Gh_A13G0831	1	385_386insGTGCA
Frame shift	Gh_A13G0918	4	340delC
Frame shift	Gh_A13G0942	8	807_808insT
Frame shift	Gh_A13G0943	15	1048delC
Frame shift	Gh_A13G0972	1	309_310insG
Frame shift	Gh_A13G0979	1	536delG
Frame shift	Gh_A13G0983	1	20_23del
Frame shift	Gh_A13G1010	3	286_287insT
Frame shift	Gh_A13G1021	1	1_2insA
Frame shift	Gh_A13G1146	2	572delA
Frame shift	Gh_A13G1156	1	385_386insA

Frame shift	Gh_A13G1234	3	298_301del
Frame shift	Gh_A13G1234	5	783delT
Frame shift	Gh_A13G1313	2	180delA
Frame shift	Gh_A13G1315	4	1287delT
Frame shift	Gh_A13G1329	2	504_505del
Frame shift	Gh_A13G1380	2	822_823insTT
Frame shift	Gh_A13G1648	2	228delT
Frame shift	Gh_A13G1656	4	653_654insAAGGTTGATGGAACCATCACCGGCTGAATCTG ATC
Frame shift	Gh_A13G1677	16	2373_2374del
Frame shift	Gh_A13G1677	25	3477_3478insG
Frame shift	Gh_A13G1677	28	3816_3817insT
Frame shift	Gh_A13G1718	1	88delT
Frame shift	Gh_A13G1752	1	9_18del
Frame shift	Gh_A13G1754	1	110delA
Frame shift	Gh_A13G1761	1	12_13insAA
Frame shift	Gh_A13G1779	11	2722_2725del
Frame shift	Gh_A13G1814	4	610_611insA
Frame shift	Gh_A13G1818	2	238_239insAAGTA
Frame shift	Gh_A13G1831	1	229_230insA
Frame shift	Gh_A13G1836	1	211delG
Frame shift	Gh_A13G1907	2	641_642del
Frame shift	Gh_A13G1940	1	1_2insA
Frame shift	Gh_A13G1971	2	37_41del
Frame shift	Gh_A13G1988	1	334_335insT
Frame shift	Gh_A13G1997	1	1delA
Frame shift	Gh_A13G2042	2	167_168insC
Frame shift	Gh_A13G2234	1	59delG
Frame shift	Gh_A13G2349	2	353_354insA
Frame shift	Gh_D01G0131	3	514_515insC
Frame shift	Gh_D01G0289	15	1474_1475insA
Frame shift	Gh_D01G0326	10	981_982insTT
Frame shift	Gh_D01G0333	2	1137delA
Frame shift	Gh_D01G0361	4	1102_1103insT
Frame shift	Gh_D01G0433	2	121_122insT
Frame shift	Gh_D01G0440	1	1141delG
Frame shift	Gh_D01G0615	1	502delA
Frame shift	Gh_D01G0639	1	582_588del
Frame shift	Gh_D01G0642	1	49_50insC
Frame shift	Gh_D01G0776	6	549delA
Frame shift	Gh_D01G0820	1	185_206del
Frame shift	Gh_D01G1028	5	697_698insT
Frame shift	Gh_D01G1031	1	33delG
Frame shift	Gh_D01G1051	2	336delA
Frame shift	Gh_D01G1143	2	93_94insA
Frame shift	Gh_D01G1161	1	507delT
Frame shift	Gh_D01G1189	2	903delT
Frame shift	Gh_D01G1265	2	531delA
Frame shift	Gh_D01G1281	4	895_896insA

Frame shift	Gh_D01G1300	1	131delT
Frame shift	Gh_D01G1305	1	225delA
Frame shift	Gh_D01G1308	5	598_599insAT
Frame shift	Gh_D01G1318	9	946_947insT
Frame shift	Gh_D01G1344	1	412delG
Frame shift	Gh_D01G1365	12	2168_2169del
Frame shift	Gh_D01G1405	1	945_946insG
Frame shift	Gh_D01G1415	Whole gene	
Frame shift	Gh_D01G1424	3	201_202insG
Frame shift	Gh_D01G1451	5	617delT
Frame shift	Gh_D01G1462	2	49_50insA
Frame shift	Gh_D01G1475	1	1020_1021insAA
Frame shift	Gh_D01G1499	1	154_155insC
Frame shift	Gh_D01G1514	6	1347delA
Frame shift	Gh_D01G1566	1	948_966del
Frame shift	Gh_D01G1602	6	528_529insA
Frame shift	Gh_D01G1769	2	464delA
Frame shift	Gh_D01G1775	1	625_626insTTTTAAAAAGGTT
Frame shift	Gh_D01G1795	1	1113delG
Frame shift	Gh_D01G1814	3	979delT
Frame shift	Gh_D01G1912	2	51_52insG
Frame shift	Gh_D01G2023	1	1_2insAACAAAAA
Frame shift	Gh_D01G2096	2	236_237del
Frame shift	Gh_D01G2168	1	1_2insAA
Frame shift	Gh_D01G2175	1	43_44insCGACAAGAAGAAGAAGAAGAAGAAGAAGA
Frame shift	Gh_D01G2187	1	1315_1322del
Frame shift	Gh_D01G2197	2	1145delG
Frame shift	Gh_D01G2202	7	890_891insTGGA
Frame shift	Gh_D01G2279	2	1244_1245insG
Frame shift	Gh_D01G2280	10	1082_1083insT
Frame shift	Gh_D01G2293	2	185_186insTAGCTTCG
Frame shift	Gh_D01G2340	1	89delG
Frame shift	Gh_D02G0059	1	454_503del
Frame shift	Gh_D02G0059	1	1084_1085insCA
Frame shift	Gh_D02G0059	1	1293_1294insA
Frame shift	Gh_D02G0090	1	1302delT
Frame shift	Gh_D02G0122	3	401delT
Frame shift	Gh_D02G0131	1	1093_1129del
Frame shift	Gh_D02G0136	1	25delG
Frame shift	Gh_D02G0156	1	50_51del
Frame shift	Gh_D02G0162	1	1019_1020del
Frame shift	Gh_D02G0171	1	630delA
Frame shift	Gh_D02G0172	1	20_21insC
Frame shift	Gh_D02G0172	2	620delA
Frame shift	Gh_D02G0172	2	75_76insG
Frame shift	Gh_D02G0173	1	101_102insT
Frame shift	Gh_D02G0195	1	302_303insCTTAAGT
Frame shift	Gh_D02G0202	1	59_60insG

Frame shift	Gh_D02G0207	9	949_964del
Frame shift	Gh_D02G0211	4	3205_3206insG
Frame shift	Gh_D02G0211	5	3398_3399insA
Frame shift	Gh_D02G0221	5	1389delA
Frame shift	Gh_D02G0232	2	1115delT
Frame shift	Gh_D02G0233	2	2456delA
Frame shift	Gh_D02G0299	2	338delC
Frame shift	Gh_D02G0299	2	659delA
Frame shift	Gh_D02G0339	1	173delC
Frame shift	Gh_D02G0339	2	1304_1305insA
Frame shift	Gh_D02G0393	1	1delA
Frame shift	Gh_D02G0451	1	194_195insA
Frame shift	Gh_D02G0469	1	52delT
Frame shift	Gh_D02G0524	1	676_677insGGTGC
Frame shift	Gh_D02G0532	1	69_70insG
Frame shift	Gh_D02G0581	1	447_447del
Frame shift	Gh_D02G0694	1	151delA
Frame shift	Gh_D02G0708	1	794_795insG
Frame shift	Gh_D02G0765	4	1385_1397del
Frame shift	Gh_D02G0769	2	1252_1253insCA
Frame shift	Gh_D02G0826	5	570_571insG
Frame shift	Gh_D02G0870	1	38_39insG
Frame shift	Gh_D02G0924	3	354delT
Frame shift	Gh_D02G0956	4	541delT
Frame shift	Gh_D02G0971	9	5307delT
Frame shift	Gh_D02G1252	2	754delA
Frame shift	Gh_D02G1284	1	387_388insA
Frame shift	Gh_D02G1368	3	246delG
Frame shift	Gh_D02G1437	1	318delC
Frame shift	Gh_D02G1494	1	344_345del
Frame shift	Gh_D02G1506	2	543_544insG
Frame shift	Gh_D02G1512	4	794_795insG
Frame shift	Gh_D02G1523	1	81delA
Frame shift	Gh_D02G1572	3	171delC
Frame shift	Gh_D02G1816	5	973_974insT
Frame shift	Gh_D02G1855	2	1515_1516insA
Frame shift	Gh_D02G1951	3	481_482del
Frame shift	Gh_D02G2072	5	3060_3084del
Frame shift	Gh_D02G2090	4	513delT
Frame shift	Gh_D02G2121	1	181_182insGG
Frame shift	Gh_D02G2171	2	281_282insT
Frame shift	Gh_D02G2198	1	1_2insA
Frame shift	Gh_D02G2229	4	335_336insC
Frame shift	Gh_D02G2248	1	1delA
Frame shift	Gh_D02G2352	2	183_184insGTCCTTGCAAGAAAGATCATGAA
Frame shift	Gh_D02G2352	3	424delG
Frame shift	Gh_D02G2377	11	959_960insT
Frame shift	Gh_D03G0061	2	73_74insCTGGTCTGAAATCGTCCTCCGCC

Frame shift	Gh_D03G0076	11	3554_3555insA
Frame shift	Gh_D03G0303	2	2929_2930insC
Frame shift	Gh_D03G0452	Whole gene	
Frame shift	Gh_D03G0599	5	359delC
Frame shift	Gh_D03G0633	3	306_307insGA
Frame shift	Gh_D03G0694	5	1741_1742insT
Frame shift	Gh_D03G0753	3	649_650insA
Frame shift	Gh_D03G1316	1	206_207insT
Frame shift	Gh_D03G1441	1	628_629insT
Frame shift	Gh_D03G1468	5	1168_1169insA
Frame shift	Gh_D03G1527	3	2446_2450del
Frame shift	Gh_D03G1568	1	1_2insA
Frame shift	Gh_D03G1573	1	342_343insTCACGCTTACC
Frame shift	Gh_D03G1596	1	252_253insT
Frame shift	Gh_D03G1693	7	611delT
Frame shift	Gh_D03G1693	9	1311delT
Frame shift	Gh_D03G1715	3	243_244insA
Frame shift	Gh_D03G1753	3	129_130insA
Frame shift	Gh_D03G1755	22	3832_3833insTGCAGTTCCAGAACTCGGA
Frame shift	Gh_D03G1758	3	569_570insATCCCAA
Frame shift	Gh_D03G1838	11	795_796insT
Frame shift	Gh_D03G1860	2	649_650insT
Frame shift	Gh_D04G0013	1	18delT
Frame shift	Gh_D04G0055	14	1313_1314insA
Frame shift	Gh_D04G0174	1	267delT
Frame shift	Gh_D04G0202	1	77delC
Frame shift	Gh_D04G0238	1	1574delC
Frame shift	Gh_D04G0255	2	2428_2429insAGTA
Frame shift	Gh_D04G0259	1	313_314insAGTA
Frame shift	Gh_D04G0336	1	768delT
Frame shift	Gh_D04G0359	1	597_598del
Frame shift	Gh_D04G0413	1	33_34insCTTA
Frame shift	Gh_D04G0419	10	1740_1741insG
Frame shift	Gh_D04G0419	4	675_676insA
Frame shift	Gh_D04G0430	1	200_201del
Frame shift	Gh_D04G0440	3	1338delC
Frame shift	Gh_D04G0501	2	300_301del
Frame shift	Gh_D04G0556	1	155_171del
Frame shift	Gh_D04G0610	7	1638_1639insG
Frame shift	Gh_D04G0656	14	2286_2289del
Frame shift	Gh_D04G0799	1	242_243insA
Frame shift	Gh_D04G0822	1	223_224insAA
Frame shift	Gh_D04G0862	1	142delG
Frame shift	Gh_D04G0877	2	179delA
Frame shift	Gh_D04G0881	2	219delG
Frame shift	Gh_D04G0881	3	867delC
Frame shift	Gh_D04G0889	2	254_255insT
Frame shift	Gh_D04G0889	3	321_327del

Frame shift	Gh_D04G0904	1	21_22insGAAAG
Frame shift	Gh_D04G0909	4	448delC
Frame shift	Gh_D04G0910	3	643_644del
Frame shift	Gh_D04G0915	1	553_593del
Frame shift	Gh_D04G0957	Whole gene	
Frame shift	Gh_D04G0977	1	368_371del
Frame shift	Gh_D04G1004	2	35_36insG
Frame shift	Gh_D04G1022	1	1017_1018insAC
Frame shift	Gh_D04G1034	4	424_425del
Frame shift	Gh_D04G1037	1	705delA
Frame shift	Gh_D04G1095	1	627delC
Frame shift	Gh_D04G1100	1	44delC
Frame shift	Gh_D04G1100	2	399_400insTGCAAAAATCCACGAGTTGT
Frame shift	Gh_D04G1142	4	1316_1317insAG
Frame shift	Gh_D04G1150	8	1300_1301insC
Frame shift	Gh_D04G1236	6	1158delT
Frame shift	Gh_D04G1249	1	224delT
Frame shift	Gh_D04G1287	5	1449_1450del
Frame shift	Gh_D04G1308	2	515_519del
Frame shift	Gh_D04G1339	2	268_269insT
Frame shift	Gh_D04G1429	1	609_610insG
Frame shift	Gh_D04G1477	3	911delA
Frame shift	Gh_D04G1477	7	1784_1785del
Frame shift	Gh_D04G1493	1	631_632insA
Frame shift	Gh_D04G1592	1	28_29insC
Frame shift	Gh_D04G1678	5	815delA
Frame shift	Gh_D04G1688	1	617_624del
Frame shift	Gh_D04G1763	7	1819delT
Frame shift	Gh_D04G1816	6	1491_1492insG
Frame shift	Gh_D04G1827	4	1106_1113del
Frame shift	Gh_D04G1838	3	834_835insCACT
Frame shift	Gh_D04G1939	1	1_2insA
Frame shift	Gh_D04G1950	1	626_645del
Frame shift	Gh_D04G1974	3	336delC
Frame shift	Gh_D04G1979	1	12_13insA
Frame shift	Gh_D04G1982	4	553_554del
Frame shift	Gh_D04G1983	2	183_184insTT
Frame shift	Gh_D04G2010	5	914_915insG
Frame shift	Gh_D04G2024	8	1483delA
Frame shift	Gh_D04G2028	1	6delA
Frame shift	Gh_D05G0097	1	1delA
Frame shift	Gh_D05G0298	1	733delA
Frame shift	Gh_D05G0298	2	888_889del
Frame shift	Gh_D05G0581	8	1029delT
Frame shift	Gh_D05G0634	6	608_609del
Frame shift	Gh_D05G1072	1	633delA
Frame shift	Gh_D05G1099	7	1380delA
Frame shift	Gh_D05G1103	6	992_993insA

Frame shift	Gh_D05G1177	2	724_725insGCCTGTCC
Frame shift	Gh_D05G1196	2	339_342del
Frame shift	Gh_D05G1441	1	350_351del
Frame shift	Gh_D05G1452	1	117delA
Frame shift	Gh_D05G1541	2	111delT
Frame shift	Gh_D05G1541	3	221delC
Frame shift	Gh_D05G1694	1	1delA
Frame shift	Gh_D05G1757	3	222delG
Frame shift	Gh_D05G1791	1	409_410insA
Frame shift	Gh_D05G1807	7	1011_1012del
Frame shift	Gh_D05G1884	1	44_45insGG
Frame shift	Gh_D05G2001	4	430_442del
Frame shift	Gh_D05G2258	2	275_276insCC
Frame shift	Gh_D05G2422	4	200_201insT
Frame shift	Gh_D05G2749	3	599_612del
Frame shift	Gh_D05G2763	1	290_291insTTTT
Frame shift	Gh_D05G2818	2	148_149insA
Frame shift	Gh_D05G2903	1	636_643del
Frame shift	Gh_D05G3181	3	755_756insG
Frame shift	Gh_D05G3196	7	1045_1045del
Frame shift	Gh_D05G3257	3	1639delT
Frame shift	Gh_D05G3277	1	213_214del
Frame shift	Gh_D05G3376	3	1917delC
Frame shift	Gh_D05G3381	1	126delA
Frame shift	Gh_D05G3473	2	891_892insT
Frame shift	Gh_D05G3486	15	1307_1308insT
Frame shift	Gh_D05G3551	1	847_848insT
Frame shift	Gh_D05G3572	1	151_152insT
Frame shift	Gh_D05G3583	1	92_93insCG
Frame shift	Gh_D05G3587	1	1221delC
Frame shift	Gh_D05G3602	3	1285delA
Frame shift	Gh_D05G3615	1	507_508del
Frame shift	Gh_D05G3631	2	533_534insCCTAGCGG
Frame shift	Gh_D05G3663	1	1569_1617del
Frame shift	Gh_D05G3667	5	374_375insG
Frame shift	Gh_D05G3670	1	2570delT
Frame shift	Gh_D05G3675	1	1delA
Frame shift	Gh_D05G3696	2	798_799del
Frame shift	Gh_D05G3696	2	1101_1116del
Frame shift	Gh_D05G3696	2	1503_1504insTCGAATGG
Frame shift	Gh_D05G3724	2	47_48insT
Frame shift	Gh_D05G3833	8	1290delG
Frame shift	Gh_D05G3864	3	746_747insT
Frame shift	Gh_D06G0096	2	1288_1289insA
Frame shift	Gh_D06G0251	4	1250_1251del
Frame shift	Gh_D06G0306	1	84delA
Frame shift	Gh_D06G0310	2	450delT
Frame shift	Gh_D06G0314	1	143delA

Frame shift	Gh_D06G0327	1	464_465insT
Frame shift	Gh_D06G0341	2	782_785del
Frame shift	Gh_D06G0363	4	245delC
Frame shift	Gh_D06G0389	1	664_665insA
Frame shift	Gh_D06G0478	1	80delG
Frame shift	Gh_D06G0512	2	151_157del
Frame shift	Gh_D06G0528	7	503_504insT
Frame shift	Gh_D06G0535	3	469delT
Frame shift	Gh_D06G0573	1	1350_1351insT
Frame shift	Gh_D06G0651	5	804_805insC
Frame shift	Gh_D06G0652	9	1219_1220insT
Frame shift	Gh_D06G0722	1	80_81insA
Frame shift	Gh_D06G0728	2	307_308insT
Frame shift	Gh_D06G0730	1	4_5insA
Frame shift	Gh_D06G0739	1	94delA
Frame shift	Gh_D06G0766	4	558_558del
Frame shift	Gh_D06G0767	6	625_626insA
Frame shift	Gh_D06G0820	3	798_799del
Frame shift	Gh_D06G0932	1	140_141insG
Frame shift	Gh_D06G0951	12	1519delC
Frame shift	Gh_D06G0952	2	2974_2975del
Frame shift	Gh_D06G0959	5	587_588del
Frame shift	Gh_D06G0961	8	730_734del
Frame shift	Gh_D06G1067	7	1393delC
Frame shift	Gh_D06G1098	2	475_476insAA
Frame shift	Gh_D06G1100	1	85delA
Frame shift	Gh_D06G1191	1	222delC
Frame shift	Gh_D06G1211	10	1926delT
Frame shift	Gh_D06G1276	2	99delG
Frame shift	Gh_D06G1295	6	1773_1774insTTGC
Frame shift	Gh_D06G1324	3	540_549del
Frame shift	Gh_D06G1324	Whole gene	
Frame shift	Gh_D06G1333	2	437_438insA
Frame shift	Gh_D06G1360	1	1197delT
Frame shift	Gh_D06G1360	1	1640delC
Frame shift	Gh_D06G1419	1	281_282del
Frame shift	Gh_D06G1471	5	897_898insG
Frame shift	Gh_D06G1491	4	293delT
Frame shift	Gh_D06G1522	6	490delT
Frame shift	Gh_D06G1530	1	222_223insT
Frame shift	Gh_D06G1557	4	587delT
Frame shift	Gh_D06G1560	10	885delA
Frame shift	Gh_D06G1560	4	367delT
Frame shift	Gh_D06G1560	5	479_480del
Frame shift	Gh_D06G1608	4	752_753insA
Frame shift	Gh_D06G1642	1	43delC
Frame shift	Gh_D06G1712	1	180delA
Frame shift	Gh_D06G1765	4	1395delG

Frame shift	Gh_D06G1849	1	603_610del
Frame shift	Gh_D06G1849	1	905_906insA
Frame shift	Gh_D06G1876	1	32delA
Frame shift	Gh_D06G1877	3	2297_2298insG
Frame shift	Gh_D06G1885	1	180_181insA
Frame shift	Gh_D06G1929	2	147_174del
Frame shift	Gh_D06G1995	1	20delA
Frame shift	Gh_D06G2031	3	1372_1381del
Frame shift	Gh_D06G2074	2	856delT
Frame shift	Gh_D06G2116	9	904_905del
Frame shift	Gh_D06G2116	9	819_820insA
Frame shift	Gh_D06G2172	1	120delC
Frame shift	Gh_D06G2174	7	1341delA
Frame shift	Gh_D06G2177	1	192_193insT
Frame shift	Gh_D06G2250	5	924_925insT
Frame shift	Gh_D06G2250	8	1831delG
Frame shift	Gh_D06G2321	2	369delT
Frame shift	Gh_D06G2321	3	428_429insG
Frame shift	Gh_D06G2343	4	465delA
Frame shift	Gh_D06G2355	2	570_571CTTG,
Frame shift	Gh_D07G0072	10	1130delA
Frame shift	Gh_D07G0076	3	239delA
Frame shift	Gh_D07G0184	2	731delG
Frame shift	Gh_D07G0326	5	716_717insGT
Frame shift	Gh_D07G0340	2	506_516del
Frame shift	Gh_D07G0385	2	999delT
Frame shift	Gh_D07G0415	9	1127delA
Frame shift	Gh_D07G0478	2	339_340insCCAG
Frame shift	Gh_D07G0517	1	470_471insA
Frame shift	Gh_D07G0587	2	1687_1688insGA
Frame shift	Gh_D07G0588	5	794_795del
Frame shift	Gh_D07G0871	9	928_929insTTAACTTATCAAGT
Frame shift	Gh_D07G0877	4	1081_1082insG
Frame shift	Gh_D07G1111	6	1275_1276insA
Frame shift	Gh_D07G1111	6	1080_1081insT
Frame shift	Gh_D07G1214	1	439_443del
Frame shift	Gh_D07G1226	1	150_151insG
Frame shift	Gh_D07G1373	8	738_739insA
Frame shift	Gh_D07G1507	2	38delA
Frame shift	Gh_D07G1705	1	552_553insC
Frame shift	Gh_D07G1709	2	127_128insCCACT
Frame shift	Gh_D07G1748	1	292_293insA
Frame shift	Gh_D07G1751	1	1909_1910insGA
Frame shift	Gh_D07G1844	1	1886delT
Frame shift	Gh_D07G1982	1	28_32del
Frame shift	Gh_D07G2039	2	1250delG
Frame shift	Gh_D07G2084	3	314_315insGT
Frame shift	Gh_D07G2106	9	1587_1588insG

Frame shift	Gh_D07G2107	2	898_899insT
Frame shift	Gh_D07G2107	3	1117_1118insA
Frame shift	Gh_D07G2107	4	1360delT
Frame shift	Gh_D07G2180	5	340_341insCAAAACGGCAGAG
Frame shift	Gh_D07G2201	1	18_19insGGGGGGG
Frame shift	Gh_D07G2253	9	3403_3407del
Frame shift	Gh_D07G2254	1	1120delT
Frame shift	Gh_D07G2264	2	556_557insT
Frame shift	Gh_D07G2271	1	1315_1316insG
Frame shift	Gh_D07G2364	3	635_636insGAGTT
Frame shift	Gh_D08G0013	6	731_732insTC
Frame shift	Gh_D08G0046	8	3255_3256insT
Frame shift	Gh_D08G0083	2	78delA
Frame shift	Gh_D08G0281	1	224_225insT
Frame shift	Gh_D08G0372	1	373_380del
Frame shift	Gh_D08G0441	2	627_628insA
Frame shift	Gh_D08G0443	2	425delT
Frame shift	Gh_D08G0445	1	157delC
Frame shift	Gh_D08G0480	11	2270delA
Frame shift	Gh_D08G0482	4	1926delA
Frame shift	Gh_D08G0487	11	1004delT
Frame shift	Gh_D08G0528	5	1122_1123del
Frame shift	Gh_D08G0555	1	1330_1331insA
Frame shift	Gh_D08G0782	6	648_649insG
Frame shift	Gh_D08G0792	1	331delG
Frame shift	Gh_D08G0860	1	84delG
Frame shift	Gh_D08G1117	4	304_305insA
Frame shift	Gh_D08G1118	1	83_84insCGACCTCA
Frame shift	Gh_D08G1127	3	646delA
Frame shift	Gh_D08G1252	5	374delA
Frame shift	Gh_D08G1529	1	9delC
Frame shift	Gh_D08G1550	2	170delA
Frame shift	Gh_D08G1551	5	1135_1136del
Frame shift	Gh_D08G1781	1	56_57insT
Frame shift	Gh_D08G1831	2	28delG
Frame shift	Gh_D08G1881	1	1517_1523del
Frame shift	Gh_D08G1993	2	545delT
Frame shift	Gh_D08G1995	10	1718_1721del
Frame shift	Gh_D08G1995	3	474_475insAA
Frame shift	Gh_D08G1995	4	771_772insT
Frame shift	Gh_D08G2016	15	7908_7909insA
Frame shift	Gh_D08G2057	1	368_369insA
Frame shift	Gh_D08G2154	1	698_699del
Frame shift	Gh_D08G2177	1	397delT
Frame shift	Gh_D08G2236	1	144_145insACAAC
Frame shift	Gh_D08G2274	5	649_655del
Frame shift	Gh_D08G2312	2	828delT
Frame shift	Gh_D08G2502	1	29_30del

Frame shift	Gh_D08G2531	2	1029_1030insA
Frame shift	Gh_D08G2593	4	595_596insT
Frame shift	Gh_D09G0030	3	471delA
Frame shift	Gh_D09G0152	1	1delA
Frame shift	Gh_D09G0162	2	688_689insTGACATG
Frame shift	Gh_D09G0173	1	1201_1202insG
Frame shift	Gh_D09G0176	1	2357_2370del
Frame shift	Gh_D09G0217	1	1526_1527del
Frame shift	Gh_D09G0219	1	385delA
Frame shift	Gh_D09G0255	2	380_381del
Frame shift	Gh_D09G0311	2	1029delT
Frame shift	Gh_D09G0354	3	360delG
Frame shift	Gh_D09G0362	1	80_81insT
Frame shift	Gh_D09G0365	1	202_203del
Frame shift	Gh_D09G0601	12	1187_1188insT
Frame shift	Gh_D09G0650	1	245_246insA
Frame shift	Gh_D09G0757	2	92_93del
Frame shift	Gh_D09G0826	1	122_123insTC
Frame shift	Gh_D09G0829	1	1_2insAA
Frame shift	Gh_D09G1027	5	756_757insATCT
Frame shift	Gh_D09G1067	1	240_241insG
Frame shift	Gh_D09G1124	2	2544delC
Frame shift	Gh_D09G1239	1	169delC
Frame shift	Gh_D09G1369	Whole gene	
Frame shift	Gh_D09G1373	10	2443delA
Frame shift	Gh_D09G1373	10	2478_2479insA
Frame shift	Gh_D09G1386	4	778delA
Frame shift	Gh_D09G1418	1	195_237del
Frame shift	Gh_D09G1440	2	1444delC
Frame shift	Gh_D09G1455	1	208delA
Frame shift	Gh_D09G1637	1	143delT
Frame shift	Gh_D09G1830	1	5delG
Frame shift	Gh_D09G1843	2	1012_1013insT
Frame shift	Gh_D09G1852	2	451delG
Frame shift	Gh_D09G1876	2	1252_1253del
Frame shift	Gh_D09G1913	2	630_631insA
Frame shift	Gh_D09G1962	12	1798_1799insC
Frame shift	Gh_D09G2136	1	562delG
Frame shift	Gh_D09G2306	1	39delC
Frame shift	Gh_D09G2356	2	469_470insT
Frame shift	Gh_D09G2408	2	382_392CCCCCCCCCCCCCTT,
Frame shift	Gh_D09G2454	1	927_928insT
Frame shift	Gh_D10G0004	1	18_19insACTTC
Frame shift	Gh_D10G0126	16	3423delC
Frame shift	Gh_D10G0256	2	87_88insC
Frame shift	Gh_D10G0267	5	516delG
Frame shift	Gh_D10G0376	3	361_373del
Frame shift	Gh_D10G0476	5	1092_1092del

Frame shift	Gh_D10G0620	2	1150_1151insAT
Frame shift	Gh_D10G0635	1	1131delA
Frame shift	Gh_D10G0657	2	199_200del
Frame shift	Gh_D10G0829	13	1309delC
Frame shift	Gh_D10G0832	5	1170_1171insTT
Frame shift	Gh_D10G1014	2	676delG
Frame shift	Gh_D10G1088	1	37_43del
Frame shift	Gh_D10G1125	1	225_226insCG
Frame shift	Gh_D10G1194	3	526_527del
Frame shift	Gh_D10G1265	1	10_11insCCCC
Frame shift	Gh_D10G1337	2	619_620del
Frame shift	Gh_D10G1407	2	229_230insT
Frame shift	Gh_D10G1911	1	264_265insC
Frame shift	Gh_D10G1941	1	1423_1424insG
Frame shift	Gh_D10G1941	1	33delC
Frame shift	Gh_D10G1943	2	2153_2154del
Frame shift	Gh_D10G1951	1	189_190del
Frame shift	Gh_D10G1957	1	1722_1723insTTGA
Frame shift	Gh_D10G2036	1	143_153del
Frame shift	Gh_D10G2093	1	1_2insA
Frame shift	Gh_D10G2127	2	350_353del
Frame shift	Gh_D10G2142	1	153delG
Frame shift	Gh_D10G2208	1	184_185insA
Frame shift	Gh_D10G2232	3	2590delC
Frame shift	Gh_D10G2250	1	1_2insAA
Frame shift	Gh_D10G2254	9	719_720insCAAA
Frame shift	Gh_D10G2263	1	86delA
Frame shift	Gh_D10G2278	1	801delA
Frame shift	Gh_D10G2281	6	4709delT
Frame shift	Gh_D10G2281	8	5839_5840del
Frame shift	Gh_D10G2317	3	794_804del
Frame shift	Gh_D10G2333	3	949_950insG
Frame shift	Gh_D10G2352	6	2351_2352insT
Frame shift	Gh_D10G2371	3	263_264insG
Frame shift	Gh_D10G2446	6	1246_1247insC
Frame shift	Gh_D10G2525	4	1827delA
Frame shift	Gh_D10G2532	7	1310_1311insAACA
Frame shift	Gh_D10G2573	7	1302_1303insG
Frame shift	Gh_D10G2587	1	374_375insCAAGACATTTGGGG
Frame shift	Gh_D10G2591	1	307delG
Frame shift	Gh_D10G2628	1	387_388insT
Frame shift	Gh_D11G0005	4	836_837insA
Frame shift	Gh_D11G0128	2	2415_2416insA
Frame shift	Gh_D11G0207	1	221_222insC
Frame shift	Gh_D11G0210	6	501_504del
Frame shift	Gh_D11G0271	9	1533_1537del
Frame shift	Gh_D11G0272	6	722delA
Frame shift	Gh_D11G0309	3	676delG

Frame shift	Gh_D11G0406	4	1914delA
Frame shift	Gh_D11G0414	9	1236_1239del
Frame shift	Gh_D11G0434	2	439_461del
Frame shift	Gh_D11G0540	1	341delT
Frame shift	Gh_D11G0648	2	159delA
Frame shift	Gh_D11G0698	2	218delA
Frame shift	Gh_D11G0706	4	1400_1401insA
Frame shift	Gh_D11G0733	4	554delT
Frame shift	Gh_D11G0776	5	805_806insG
Frame shift	Gh_D11G0783	2	324_325del
Frame shift	Gh_D11G0799	1	748delC
Frame shift	Gh_D11G0854	2	421_422insAT
Frame shift	Gh_D11G0857	1	12delA
Frame shift	Gh_D11G0859	2	43delA
Frame shift	Gh_D11G0865	1	673delA
Frame shift	Gh_D11G0903	1	62_63insA
Frame shift	Gh_D11G0903	4	1485_1501del
Frame shift	Gh_D11G0931	1	118_119insA
Frame shift	Gh_D11G0968	10	3084delC
Frame shift	Gh_D11G0982	1	44_45insA
Frame shift	Gh_D11G0982	14	1844_1845insG
Frame shift	Gh_D11G1004	8	1026_1027insA
Frame shift	Gh_D11G1021	1	1275_1276del
Frame shift	Gh_D11G1084	16	3226_3227del
Frame shift	Gh_D11G1119	7	1600_1603del
Frame shift	Gh_D11G1119	7	1066_1067del
Frame shift	Gh_D11G1127	1	231_232insG
Frame shift	Gh_D11G1135	1	757delA
Frame shift	Gh_D11G1193	2	137_138del
Frame shift	Gh_D11G1227	4	534_535insA
Frame shift	Gh_D11G1296	3	216_217del
Frame shift	Gh_D11G1513	2	146_150del
Frame shift	Gh_D11G1514	3	217_218insAA
Frame shift	Gh_D11G1602	1	1_2insA
Frame shift	Gh_D11G1609	1	272_284del
Frame shift	Gh_D11G1646	1	119_120insCCCTATTCCC
Frame shift	Gh_D11G1676	1	183_184del
Frame shift	Gh_D11G1812	17	1820_1821del
Frame shift	Gh_D11G1855	1	147_148insCCCC
Frame shift	Gh_D11G1950	1	1050_1051insG
Frame shift	Gh_D11G2091	12	1671delA
Frame shift	Gh_D11G2095	1	155_156insT
Frame shift	Gh_D11G2137	5	547delG
Frame shift	Gh_D11G2146	2	707_708insAT
Frame shift	Gh_D11G2452	3	376delC
Frame shift	Gh_D11G2558	1	65delC
Frame shift	Gh_D11G2589	6	529delA
Frame shift	Gh_D11G2672	5	4847_4848del

Frame shift	Gh_D11G2741	2	76delG
Frame shift	Gh_D11G2741	2	114delA
Frame shift	Gh_D11G2763	1	98delA
Frame shift	Gh_D11G2918	1	47delT
Frame shift	Gh_D11G2941	1	867_868insGCTA
Frame shift	Gh_D11G2950	2	2590_2593del
Frame shift	Gh_D11G2959	1	216_229del
Frame shift	Gh_D11G2964	2	1158_1162del
Frame shift	Gh_D11G3006	6	747_748insA
Frame shift	Gh_D11G3069	1	188_189insA
Frame shift	Gh_D11G3107	14	11084delT
Frame shift	Gh_D11G3108	13	8121_8122insT
Frame shift	Gh_D11G3113	2	2639_2646del
Frame shift	Gh_D11G3113	6	3450_3451insA
Frame shift	Gh_D11G3115	2	855delC
Frame shift	Gh_D11G3117	2	83_84insA
Frame shift	Gh_D11G3234	12	1998_2007del
Frame shift	Gh_D11G3237	5	1026_1027insA
Frame shift	Gh_D11G3320	2	966_969del
Frame shift	Gh_D11G3398	1	488_489del
Frame shift	Gh_D11G3402	6	1155_1156insT
Frame shift	Gh_D11G3413	2	373delG
Frame shift	Gh_D11G3529	1	300_309del
Frame shift	Gh_D12G0100	1	1_2insA
Frame shift	Gh_D12G0117	2	419delA
Frame shift	Gh_D12G0136	1	42_43insCCCCT
Frame shift	Gh_D12G0420	15	1694delC
Frame shift	Gh_D12G0424	1	115delA
Frame shift	Gh_D12G0528	1	185_186insT
Frame shift	Gh_D12G0545	4	163_164del
Frame shift	Gh_D12G0571	2	823_824insA
Frame shift	Gh_D12G0575	3	398_402del
Frame shift	Gh_D12G0926	2	500_501insA
Frame shift	Gh_D12G0952	4	415_418del
Frame shift	Gh_D12G1086	1	724_730del
Frame shift	Gh_D12G1301	1	24_25insACAAC
Frame shift	Gh_D12G1344	1	83delG
Frame shift	Gh_D12G1367	2	1777_1778insA
Frame shift	Gh_D12G1403	5	392_393insT
Frame shift	Gh_D12G1455	1	3_6del
Frame shift	Gh_D12G1458	10	1526delC
Frame shift	Gh_D12G1476	3	831_832insT
Frame shift	Gh_D12G1685	2	549delG
Frame shift	Gh_D12G1726	4	278_281del
Frame shift	Gh_D12G1821	1	214delG
Frame shift	Gh_D12G1837	2	1112_1113insG
Frame shift	Gh_D12G1840	1	11delA
Frame shift	Gh_D12G1898	7	1123_1124insAA

Frame shift	Gh_D12G2020	7	713_714insTT
Frame shift	Gh_D12G2023	2	1750_1751insG
Frame shift	Gh_D12G2062	1	162_165del
Frame shift	Gh_D12G2062	2	363_364insT
Frame shift	Gh_D12G2137	17	1421delT
Frame shift	Gh_D12G2528	1	210delT
Frame shift	Gh_D12G2652	2	417_421del
Frame shift	Gh_D12G2664	5	504delA
Frame shift	Gh_D12G2670	1	1_2insA
Frame shift	Gh_D12G2780	2	745_746insTTATT
Frame shift	Gh_D13G0084	2	460_461insCTTA
Frame shift	Gh_D13G0085	1	78_79insC
Frame shift	Gh_D13G0085	2	189_196del
Frame shift	Gh_D13G0085	3	314delG
Frame shift	Gh_D13G0085	5	653_654insA
Frame shift	Gh_D13G0085	7	808_809insC
Frame shift	Gh_D13G0088	6	473delT
Frame shift	Gh_D13G0104	3	550_551insAG
Frame shift	Gh_D13G0133	11	1422delT
Frame shift	Gh_D13G0134	11	1785_1786insT
Frame shift	Gh_D13G0134	2	399_400insA
Frame shift	Gh_D13G0134	7	1244_1245insA
Frame shift	Gh_D13G0239	1	405delG
Frame shift	Gh_D13G0520	1	1_2insA
Frame shift	Gh_D13G0543	2	329_332del
Frame shift	Gh_D13G0544	2	1781_1782insG
Frame shift	Gh_D13G0572	1	131_134del
Frame shift	Gh_D13G0607	2	896_897insT
Frame shift	Gh_D13G0659	2	338_339insT
Frame shift	Gh_D13G0669	1	103delA
Frame shift	Gh_D13G0691	3	126_133del
Frame shift	Gh_D13G0729	2	90delA
Frame shift	Gh_D13G0769	Whole gene	
Frame shift	Gh_D13G0842	2	234delT
Frame shift	Gh_D13G0864	1	1_2insA
Frame shift	Gh_D13G0879	2	135delG
Frame shift	Gh_D13G0951	3	387_388insA
Frame shift	Gh_D13G1122	2	692delG
Frame shift	Gh_D13G1226	4	341delG
Frame shift	Gh_D13G1226	4	421_422insAGGTA
Frame shift	Gh_D13G1272	1	1_2insAAAA
Frame shift	Gh_D13G1300	2	356_359del
Frame shift	Gh_D13G1335	11	826delC
Frame shift	Gh_D13G1373	12	1483delA
Frame shift	Gh_D13G1425	7	858delG
Frame shift	Gh_D13G1455	6	1348delG
Frame shift	Gh_D13G1460	3	594_595insC
Frame shift	Gh_D13G1570	1	1619_1620insA

Frame shift	Gh_D13G1660	1	237_238del
Frame shift	Gh_D13G1727	10	842delG
Frame shift	Gh_D13G1743	1	1065_1066insT
Frame shift	Gh_D13G1743	1	799_811del
Frame shift	Gh_D13G1743	1	64_65insGGATGCTTTCACC
Frame shift	Gh_D13G1891	2	235delG
Frame shift	Gh_D13G1980	1	161_162insA
Frame shift	Gh_D13G2124	3	803delC
Frame shift	Gh_D13G2126	1	1_2insA
Frame shift	Gh_D13G2159	1	146_156del
Frame shift	Gh_D13G2162	1	338_339insTACTC
Frame shift	Gh_D13G2185	26	2825_2826del
Frame shift	Gh_D13G2209	1	356_357insC
Frame shift	Gh_D13G2291	1	266delC
Frame shift	Gh_D13G2319	1	36delG
Frame shift	Gh_D13G2322	11	1687_1715del
Frame shift	Gh_D13G2352	2	300_306del
Frame shift	Gh_D13G2380	4	709_727del
Frame shift	Gh_D13G2480	2	2515_2516insC
Frame shift	Gh_D13G2494	1	37_38insCCCGCCGTTACAA
Frame shift	Gh_D13G2507	1	926_927insGGTGGTGGAT
Frame shift	Gh_D13G2527	9	1905_1906insA
Frame shift	Gh_Sca004802G01	6	1247delA
Frame shift	Gh_Sca004822G02	1	356_357insC
Frame shift	Gh_Sca004831G03	8	1322delA
Frame shift	Gh_Sca004865G01	1	154delC
Frame shift	Gh_Sca004871G05	1	480_481insCCCAAACCCCAACC
Frame shift	Gh_Sca004909G04	Whole gene	
Frame shift	Gh_Sca004925G03	2	776delT
Frame shift	Gh_Sca004952G01	2	232_232GGTTTTTTA,
Frame shift	Gh_Sca004965G03	5	3060_3061del
Frame shift	Gh_Sca004965G03	8	4403delT
Frame shift	Gh_Sca005047G02	3	211_212insGTTGCAA
Frame shift	Gh_Sca005111G02	2	92delA
Frame shift	Gh_Sca005161G01	Whole gene	
Frame shift	Gh_Sca005232G02	1	206_207insT
Frame shift	Gh_Sca005718G01	11	1924_1925del
Frame shift	Gh_Sca005755G01	4	2626delG
Frame shift	Gh_Sca007061G01	2	1835_1836insT
Frame shift	Gh_Sca008811G01	2	319delG
Frame shift	Gh_Sca011476G01	1	365_405del
Frame shift	Gh_Sca013434G01	7	1016_1017insAG
Frame shift	Gh_Sca013939G01	3	412_419del
Frame shift	Gh_Sca014641G01	4	226_227insTGGTTGTTGG
Frame shift	Gh_Sca014817G01	1	302delA
Frame shift	Gh_Sca026213G01	1	476delC
Frame shift	Gh_Sca033921G01	2	298delG
Frame shift	Gh_Sca109475G01	1	243delA

Stop gain	Gh_A01G0632	1	12_13insGAATGATGAATGAT
Stop gain	Gh_A01G1900	6	481_482insGAATCTTATCATGTCAGGTTGCTGAAGATTCGT
Stop gain	Gh_A02G1530	1	1439delC
Stop gain	Gh_A03G0076	3	1126_1127insGGAAACTACCTGGGTGATT
Stop gain	Gh_A05G0640	1	1delA
Stop gain	Gh_A05G0960	1	36_37insTGTCTCTAGAAGGCTATGAT
Stop gain	Gh_A06G0490	4	185delG
Stop gain	Gh_A06G1102	2	506_507insCTTTAGCGGCGTCTTATAAA
Stop gain	Gh_A07G1322	4	1381delA
Stop gain	Gh_A07G1353	1	233delG
Stop gain	Gh_A07G1976	3	721_722insCTCACTA
Stop gain	Gh_A08G0640	2	690_691insAGGAGTTG
Stop gain	Gh_A09G1205	7	2191delC
Stop gain	Gh_A10G1445	1	94delA
Stop gain	Gh_A11G0202	7	1873_1874insAA
Stop gain	Gh_A11G0899	1	556delC
Stop gain	Gh_A11G1360	1	1delA
Stop gain	Gh_A11G1363	2	347_348insAGACTTAAACCAGACTTCATAGACTTCAT
Stop gain	Gh_A12G0388	1	406delA
Stop gain	Gh_A12G0922	7	2218_2219insA
Stop gain	Gh_A12G2081	7	1166delT
Stop gain	Gh_A12G2593	1	1delA
Stop gain	Gh_A13G1536	6	1287_1288insGGATAAGATCCAGCAGTTTCCGCCAGAGGCAC
Stop gain	Gh_A13G1677	19	2659_2660insCATAAT
Stop gain	Gh_D02G0149	2	1097_1098insTGCATAG
Stop gain	Gh_D02G0581	1	190delA
Stop gain	Gh_D03G1096	1	32_33insCAATTAGACCAGT
Stop gain	Gh_D04G0016	1	27_28insT
Stop gain	Gh_D04G0244	1	394_395insCTTCCTCCATATAATTCAAGT
Stop gain	Gh_D06G0630	1	1delA
Stop gain	Gh_D06G0711	9	2043_2044insTTCTAGGG
Stop gain	Gh_D06G1233	10	3546_3547insTACAGCAGCTAGGCTGCCATATTTTGATGGT
Stop gain	Gh_D06G1676	3	474_475insTAGAACAGCATCA
Stop gain	Gh_D07G0776	2	1346delT
Stop gain	Gh_D07G1810	3	1066_1067insA
Stop gain	Gh_D08G0879	3	269_270insTCATTGCTAAGAA
Stop gain	Gh_D10G0439	2	721delT
Stop gain	Gh_D10G0617	1	143delT
Stop gain	Gh_D11G0610	1	423_424insTGATGGTAAGTGATGGA
Stop gain	Gh_D11G0839	1	26_27insG
Stop gain	Gh_D11G1034	2	146_147insGTTTTTTA
Stop gain	Gh_D11G3133	1	227_228insGCTGCAGTAA
Stop gain	Gh_D12G0713	3	1248_1249insTGA
Stop gain	Gh_D13G0478	2	808delA
Stop gain	Gh_D13G1679	2	229_230insAAATCCACTCCTTTATT
Stop gain	Gh_D13G1710	1	269_270insAGAAACTTAAA

Stop loss	Gh_A01G1372	2	556delT
Stop loss	Gh_A06G0080	17	1782delA
Stop loss	Gh_A07G0204	9	887delA
Stop loss	Gh_A11G1007	3	765_766insA
Stop loss	Gh_A11G1683	5	1302delA
Stop loss	Gh_D01G1775	1	1037delA
Stop loss	Gh_D02G0127	14	3509delA
Stop loss	Gh_D02G0166	1	1376delA
Stop loss	Gh_D06G1100	1	485delA
Stop loss	Gh_D07G2341	3	737delA
Stop loss	Gh_D08G2060	4	903delG
Stop loss	Gh_D11G3220	3	609delA
Stop loss	Gh_D13G0343	1	586_587insTAAGT
Stop loss	Gh_Sca005714G01	22	5132_5133insC
