# Supplementary material for: Abundant Small Genetic Alterations after Upland Cotton Domestication
Source: Biomed Res Int. 2018 Dec 18;2018:9254302. doi: 10.1155/2018/9254302 (PMC6312614; doi:10.1155/2018/9254302)
Supplement: Supplementary 3 — Table S3: cis-acting regulatory element variations in the upstream and downstream 2-kb regions of five genes between the cultivated and wild cottons. [file 9254302.f3.pdf]

TABLE S3: *Cis*-acting regulatory element variations in the upstream and downstream 2-kb regions of five genes between the cultivated and wild cottons

No	Region	Chr	Location	SNP/Indel		*Cis*- element	Function	*Cis*- element	Function
				TM1	Tx2094	TM1		Tx2094	
**Glycerol-3-phosphate acyltransferase 3: Gh_A06G0327**									
1	Upstream	A06	4926006	A	G			CAAT-box	Common *cis*-acting element in promoter and enhancer regions
2	Upstream	A06	4926062	T	C				
3	Upstream	A06	4926534	C	T	CAAT-box	Common *cis*-acting element in promoter and enhancer regions	CAAT-box	Common *cis*-acting element in promoter and enhancer regions
1	Upstream	A06	4925722	-	AGCAA				
2	Upstream	A06	4926440	-	A	TATA-box	Core promoter element around -30 of transcription start	TATA-box	Core promoter element around -30 of transcription start
3	Upstream	A06	4927020	A	-				
4	Downstream	A06	4929938	CA	-	P-box	Gibberellin-responsive element		
5	Downstream	A06	4930395	-	A			AAGAA-motif	Found in *Avena sativa*, unknown
6	Downstream	A06	4931274	T	-	TATA-box	Core promoter element around -30 of transcription start		
7	Downstream	A06	4931460	-	AT	CAAT-box	Common *cis*-acting element in promoter and enhancer regions	CAAT-box	Common *cis*-acting element in promoter and enhancer regions
**Glycerol-3-phosphate acyltransferase 3: Gh_D06G0354**									
1	Upstream	D06	4860490	A	T			CAAT-box	Common *cis*-acting element in promoter and enhancer regions
2	Upstream	D06	4860520	C	G				
3	Upstream	D06	4860787	C	A				
4	Upstream	D06	4860863	G	T	CAAT-box	Common *cis*-acting element in promoter and enhancer regions		
5	Upstream	D06	4861360	A	G				
6	Upstream	D06	4861628	C	G				
7	Upstream	D06	4861714	T	C				
8	Upstream	D06	4861835	G	A				

9	Upstream	D06	4861851	A	G	CAAT-box	Common *cis*-acting element in promoter and enhancer regions		
10	Downstream	D06	4864979	A	G				
11	Downstream	D06	4865113	A	G	TATA-box	Core promoter element around -30 of transcription start		
12	Downstream	D06	4865117	T	A	TATA-box	Core promoter element around -30 of transcription start		
1	Downstream	D06	4861080	-	T	TATA-box	Core promoter element around -30 of transcription start	TATA-box	Core promoter element around -30 of transcription start
2	Downstream	D06	4861126	-	T	TATA-box	Core promoter element around -30 of transcription start	TATA-box	Core promoter element around -30 of transcription start
3	Downstream	D06	4861222	A	-				
4	Upstream	D06	4861486	A	-	TATA-box	Core promoter element around -30 of transcription start	TATA-box	Core promoter element around -30 of transcription start

O-methyltransferase 1: Gh_A12G1059

1	Upstream	A12	62933035	T	C	TATA-box	Core promoter element around -30 of transcription start		
2	Upstream	A12	62933400	T	C			Unnamed_4	found in *Petroselinum hortense*, unknown
3	Upstream	A12	62933441	C	T	CGTCA-motif	*Cis*-acting regulatory element involved in the MeJA-responsiveness	CAAT-box	Common *cis*-acting element in promoter and enhancer regions
4	Upstream	A12	62933562	A	G				
5	Upstream	A12	62933571	A	G	CAAT-box	Common *cis*-acting element in promoter and enhancer regions		
6	Upstream	A12	62934066	C	T			CAAT-box	Common *cis*-acting element in promoter and enhancer regions
7	Upstream	A12	62934534	A	G				

O-methyltransferase 1: Gh_D12G1183

1	Upstream	D12	39228033	C	G			GT1-motif	Light responsive element
2	Upstream	D12	39228034	G	T				
3	Upstream	D12	39228864	C	T			Sp1	Light responsive element

4	Upstream	D12	39229504	A	C	TATA-box	Core promoter element around -30 of transcription start		
5	Upstream	D12	39229872	A	C	CAAT-box	Common *cis*-acting element in promoter and enhancer regions		
1	Downstream	D12	39229174	CTT	-				

Profilin 1: Gh_A05G0193

1	Downstream	A05	2017536	T	C	AT-rich element	Binding site of AT-rich DNA binding protein (ATBP-1)		
1	Downstream	A05	2017605	TCTTTT	-	5UTR Py-rich stretch	*Cis*-acting element conferring high transcription levels		
2	Downstream	A05	2019245	ACAGACT ATAAAAA	-	TATA-box	Core promoter element around -30 of transcription start		
3	Upstream	A05	2022130	-	TA			TA-rich region	Enhancer

Profilin 1: Gh_D05G0266

1	Downstream	D05	2387240	A	G				
2	Downstream	D05	2387438	T	A			TATA-box	Core promoter element around -30 of transcription start
3	Downstream	D05	2387681	G	T				
4	Downstream	D05	2388753	C	T	Sp1	Light responsive element		
5	Upstream	D05	2389954	G	C				
6	Upstream	D05	2390043	T	A	Skn-1_motif	*Cis*-acting regulatory element required for endosperm expression	Box-W1	Fungal elicitor responsive element
7	Upstream	D05	2390115	T	A	CAAT-box	Common *cis*-acting element in promoter and enhancer regions	CAAT-box	Common *cis*-acting element in promoter and enhancer regions
8	Upstream	D05	2390192	A	T	TATA-box	Core promoter element around -30 of transcription start	TATA-box	Core promoter element around -30 of transcription start
9	Upstream	D05	2390231	G	A	CAAT-box	Common *cis*-acting element in promoter and enhancer regions	CAAT-box	Common *cis*-acting element in promoter and enhancer regions
10	Upstream	D05	2390272	C	A				
11	Upstream	D05	2390341	G	A			TATA-box	Core promoter element around

									-30 of transcription start
12	Upstream	D05	2390346	G	T				
13	Upstream	D05	2390433	G	A				
14	Upstream	D05	2390877	G	T			TATA-box	Core promoter element around -30 of transcription start
15	Upstream	D05	2391219	T	C	TATA-box	Core promoter element around -30 of transcription start		
16	Upstream	D05	2391233	T	G	ARE	*Cis*-acting regulatory element essential for the anaerobic induction		
17	Upstream	D05	2391539	C	T			TATA-box	Core promoter element around -30 of transcription start
18	Upstream	D05	2391585	C	T				
19	Upstream	D05	2391587	C	T				Common *cis*-acting element in promoter and enhancer regions
20	Upstream	D05	2391603	G	A				
21	Upstream	D05	2391627	C	T			TATA-box	Core promoter element around -30 of transcription start
1	Downstream	D05	2388235	A	-				
2	Upstream	D05	2390458	-	GTCGATT TTAACAG TCCAGTG ACTTATC ATAAAAT			+	Core promoter element around -30 of transcription start
3	Upstream	D05	2390862	-	T				
4	Upstream	D05	2390933	C	-				
5	Upstream	D05	2391574	-	TA			TATA-box	Core promoter element around -30 of transcription start

Aldehyde dehydrogenase 7: Gh_D06G1578

1	Downstream	D06	52815990	G	A			Box I	Light responsive element
2	Downstream	D06	52816170	A	C			LTR	*Cis*-acting element involved in low-temperature

									responsiveness
3	Downstream	D06	52816276	G	A			TATA-box	Core promoter element around -30
4	Downstream	D06	52816384	C	T	CAAT-box	Common *cis*-acting element in promoter and enhancer regions		
5	Downstream	D06	52816937	C	T	CCAAT-box	MYBHv1 binding site		
6	Downstream	D06	52817185	T	A				
7	Downstream	D06	52817444	T	C				
8	Upstream	D06	52822990	C	A				
9	Upstream	D06	52823767	A	G	G-box	*Cis*-acting regulatory element involved in light responsiveness		
10	Upstream	D06	52824126	A	C			Skn-1_motif	*Cis*-acting regulatory element required for endosperm expression
11	Upstream	D06	52824380	G	T			CAAT-box	Common *cis*-acting element in promoter and enhancer regions
12	Upstream	D06	52824602	C	T	Box I	Light responsive element	TATA-box	Core promoter element around -30 of transcription start
						CAAT-box	Common *cis*-acting element in promoter and enhancer regions		
						ERE	ethylene-responsive element		
13	Upstream	D06	52824822	G	A	Unnamed_4	Found in *Petroselinum hortense*, unknown		
1	Downstream	D06	52816087	-	A				
2	Downstream	D06	52816245	A	-	TATA-box	Core promoter element around -30 of transcription start	TATA-box	Core promoter element around -30 of transcription start
3	Downstream	D06	52816429	-	A				
4	Upstream	D06	52823480	-	T				
5	Upstream	D06	52824474	T	-				

Tyrosine transaminase family protein: Gh_D13G1562

1	Upstream	D13	47977576	A	G				
2	Upstream	D13	47977578	G	C				
3	Upstream	D13	47978181	C	T	MBS	MYB binding site involved in drought-inducibility	CAAT-box	Common *cis*-acting element in promoter and enhancer regions
4	Upstream	D13	47984631	T	G	TATA-box	Core promoter element around -30 of transcription start		
5	Upstream	D13	47984728	G	T				
6	Upstream	D13	47984764	T	C				
7	Upstream	D13	47984775	C	T			CAAT-box	Common *cis*-acting element in promoter and enhancer regions
8	Upstream	D13	47984831	C	T			CAAT-box	Common *cis*-acting element in promoter and enhancer regions
9	Upstream	D13	47984989	A	C				
10	Upstream	D13	47985123	G	A			Box-W1	Fungal elicitor responsive element
11	Upstream	D13	47985145	G	T			CAAT-box	Common *cis*-acting element in promoter and enhancer regions
12	Upstream	D13	47985688	T	C	TATA-box	Core promoter element around -30 of transcription start	CAAT-box	Common *cis*-acting element in promoter and enhancer regions
13	Upstream	D13	47985691	T	C	TATA-box	Core promoter element around -30 of transcription start		
1	Upstream	D13	47984385	-	T	TATA-box	Core promoter element around -30 of transcription start	TATA-box	Core promoter element around -32 of transcription start
2	Upstream	D13	47985548	-	TTTATGA T			CAAT-box	Common *cis*-acting element in promoter and enhancer regions
3	Upstream	D13	47985628	-	T	TATA-box	Core promoter element around -30 of transcription start	TATA-box	Core promoter element around -30 of transcription start
4	Upstream	D13	47986310	-	A				

Blue color :changed *cis*-elements; Green color: unchanged *cis*-elements; Grey: no *cis*-element.
